# Unveiling the Ro60-Ro52 complex

**DOI:** 10.17179/excli2024-7141

**Published:** 2024-06-10

**Authors:** Laura R. Rodríguez, Jesus Vicente de Julián-Ortiz, Fernando Rubio de la Rúa, Augusto Juste-Dolz, Ángel Maquieira, Haydar A. Mohammad-Salim, Sofiane Benmetir, Federico V. Pallardó, Pilar González-Cabo, David Gimenez-Romero

**Affiliations:** 1 Department of Physiology, Faculty of Medicine and Dentistry. University of Valencia-INCLIVA, 46010 Valencia, Spain; 2 CIBER Rare Diseases (CIBERER), 46010 Valencia, Spain; 3 Present address: Stem Cells, Aging and Neurodegeneration Group, Department of Experimental Medical Science, Faculty of Medicine, Lund Stem Cell Center, Lund University, 22184, Lund, Sweden; 4 Molecular Topology and Drug Design Research Unit, Department of Physical Chemistry, Faculty of Pharmacy, University of Valencia, Av. Vicent Andrés Estellés s/n, 46100 Valencia, Spain; 5 Department of Physical Chemistry, Faculty of Chemistry, University of Valencia, C/ Doctor Moliner 50, 46100, Burjassot, Spain; 6 Center for Research and Innovation on Bioengineering (Ci2B), Universitat Politècnica de València, Camino de Vera s/n, 46022 Valencia, Spain; 7 Departamento de Química, Polytechnic University of Valencia, Camino de Vera s/n 46022, Valencia, Spain; 8 Instituto Interuniversitario de Investigación de Reconocimiento Molecular y Desarrollo Tecnológico (IDM), Universitat Politècnica de València, Universitat de València, Camino de Vera s/n, 46022 Valencia, Spain; 9 Faculty of Science, Department of Chemistry, University of Zakho, Zakho, Duhok 42001, Kurdistan Region, Iraq; 10 Process and Environmental Engineering Laboratory (LIPE), Faculty of Chemistry, University of Science and Technology of Oran Mohamed BOUDIAF, P.O. Box 1503, El Mnaouer, 31000 Oran, Algeria

**Keywords:** Ro52/Trim21, Ro60/Trove2, transient complex, QCM-D, PLA, IIF

## Abstract

The coexistence within a subcellular complex of inter-cellular proteins Ro60, responsible for preserving ncRNA quality, and Ro52, involved in intracellular proteolysis, has been a subject of ongoing debate. Employing molecular docking in tandem with experimental methods like Quartz Crystal Microbalance with Dissipation (QCM-D), Proximity Ligation Assay (PLA), and Indirect Immunofluorescence (IIF), we reveal the presence of Ro60 associating with Ro52 within the cytoplasm. This result unveils the formation of a weak transient complex with a K_a_ ≈ (3.7 ± 0.3) x 10^6^ M^-1^, where the toroid-shaped Ro60 structure interacts with the Ro52's Fc receptor, aligning horizontally within the PRY-SPRY domains of the Ro52's homodimer. The stability of this complex relies on the interaction between Ro52 chain A and specific Ro60 residues, such as K133, W177, or L185, vital in the Ro60-YRNA bond. These findings bridge the role of Ro60 in YRNA management with Ro52's function in intracellular proteolysis, emphasizing the potential impact of transient complexes on cellular pathways.

See also the graphical abstract(Fig. 1).

## Introduction

Anti‐SSA/Ro autoantibodies serve as crucial serological markers across a spectrum of systemic autoimmune diseases, encompassing idiopathic inflammatory myopathies, systemic lupus erythematosus (SLE), Sjögren's syndrome, neonatal lupus, and primary biliary cirrhosis (Franceschini and Cavazzana, 2005[20]; Yoshimi et al., 2012[46]). Nonetheless, the pathological role of these antibodies is still poorly understood. These antinuclear antibodies target the Ro/SSA antigen, initially identified by Anderson et al. in 1962. The designation "Ro" originated from a systemic lupus erythematosus patient ("Ro"), while SS-A denotes Sjogren Syndrome Type A Antigen (Alspaugh and Tan, 1975[2]).

In 1981, Steitz et al. demonstrated the formation of Ro-ribonucleoproteins (Ro-RNP) with small cytoplasmic RNAs (hY-RNA) (Lerner et al., 1981[32]), identifying two key components as Ro proteins: Ro60/TROVE2 (60 kDa) and Ro52/TRIM21 (52 kDa). Ro60, discovered in 1984, was found within a ribonucleoprotein complex with hY-RNA (Wolin and Steitz, 1984[45]), while Ro52 was later defined as part of the Ro/SSA antigen (Benchetrit et al., 1988[7]). Initial suggestions hinted at complex formation between Ro52 and Ro60; however, subsequent research failed to validate this direct interaction (Boire et al., 1995[9]; Kelekar et al., 1994[27]).

Apart from this, the intracellular localization of Ro60 and Ro52 differs significantly. Ro60 predominantly localizes within the nucleus, existing as a free protein or within a ribonucleoprotein complex (Boccitto and Wolin, 2019[8]), while shuttling between the nucleus and cytoplasm in vertebrate cells (Sim et al., 2012[40]). Conversely, Ro52 primarily resides in the cytoplasm, with a minor presence in the nucleus (Arase et al., 2022[4]). These functionally distinct proteins, encoded by separate genes on different chromosomes, pose an intriguing conundrum regarding the intimate association observed between the two autoantibodies.

Ro60 plays a critical role in binding misfolded noncoding RNAs' single-stranded ends, participating in RNA quality control, and priming it for degradation (Sim and Wolin, 2011[39]). Structurally, Ro60 assumes a toroid-shaped monomeric ring formed by antiparallel α-helical repeats and a von Willebrand factor A domain (vWFA) (Boccitto and Wolin, 2019[8]). The vWFA domain, commonly implicated in numerous multiprotein complexes, facilitates protein-protein interactions through a metal ion-dependent adhesion site (MIDAS). 

On the other hand, Ro52 operates as a ubiquitin E3 ligase, involved in degrading cytosolic virus-antibody complexes (Foss et al., 2019[18]). It is a cytosolic fragment crystallizable region (Fc) receptor, displaying the highest affinity for these fragments among human proteins. This protein acts as a liaison between the cellular self-defense system and adaptive immunity (Randow et al., 2013[36]), showcasing potential therapeutic applications such as TRIM-Away, anti-viral effector functions, and Tau degradation (Foss et al., 2019[18]). Structurally, Ro52 features four domains: An N-terminal RING, a type 2 B-box, a coiled-coil domain, and a C-terminal substrate-binding PRY-SPRY domain. It forms a homodimer in solution, binding to the Fc region of immunoglobulins through two symmetric PRY-SPRY domains (Mallery et al., 2010[33]). Its RING domain mediates E3 ubiquitin ligase activity, enabling a dual response.

Gaining insight into protein interactions is crucial for grasping their roles within cells. Methods such as proximity ligation assay (PLA), indirect immunofluorescence (IIF), and quartz crystal microbalance (QCM) aid in detecting these interactions, both within cellular environments and in controlled laboratory conditions (do Nascimento et al., 2017[15]). In this study, these methodologies will be utilized to confirm the presence of the Ro60-Ro52 complex. Computational analyses will validate the presence of this complex, providing insights into its structure. These findings could shed light on how these proteins are involved in the pathogenesis of systemic autoimmune diseases, unveiling potential therapeutic applications.

## Materials and Methods

### QCM-D assays

In our *in vitro* study of protein-protein interactions, variations in frequency (Δf) were monitored using a Q-Sense E1 device (5 MHz, QSX 301, Biolin Scientific, Gothenburg, Sweden) equipped with a liquid flow cell setup. All experiments were conducted in phosphate-buffered saline (1x PBS, 10 mM phosphate, 137 mM NaCl, 2.7 mM NaCl, pH 7.4) at a flow rate of 50 µL/min and 25 °C (do Nascimento et al., 2017[15]).

The Self-Assembly Monolayer was formed by immersing QCM-D chips in 10 mM 3-mercaptopropionic acid (Sigma-Aldrich, San Luis, USA) overnight. Subsequently, they were treated with N-ethyl-N'-(3-dimethylaminopropyl) carbodiimide (EDC, Sigma-Aldrich, San Luis, USA, purum grade)/N-hydroxysulfosuccin-imide (NHS 98 %, Sigma-Aldrich, San Luis, USA), 46 mM, for 60 min. After immersing in EDC/NHS mixture, the chips were treated with 5 mM carbohydrazide 98 % (Sigma-Aldrich, San Luis, USA), followed by the application of 100 µL of Ro60 protein (recombinant human protein, Deltaclon, Madrid, Spain) with a concentration of 33 mg/L onto the treated substrate for 60 min.

### Cell culture

Hela cells, acquired from ATCC, were cultured in DMEM high glucose (Thermo Fisher Scientific, Waltham, USA) supplemented with 10 % fetal bovine serum (FBS), 1 % L-glutamine, and 1 % Penicillin/Streptomycin. The cells were incubated at 37 °C in a humidity chamber at 5 % CO_2_ atmosphere.

### Immunofluorescence assays

HeLa cells were seeded on 13 mm Ø glass slides within a 24-well plate. Following fixation with 4 % paraformaldehyde in PBS for 10 minutes, cells underwent three 5-minute washes with PBS and were permeabilized using 0.5 % PBS-Triton-X for 10 minutes. Subsequently, the cells underwent three 5-minute washes with 0.05 % PBS-Triton and were blocked with a solution containing 10 % FBS and 0.01 % Triton for 1 hour at room temperature. A combination of primary antibodies (52 kDa Ro/SSA, sc-25351, Santa Cruz, Heidelberg, Germany; Ro60/SS-A, ab219973, Abcam, Cambridge, UK) was then incubated overnight at 4 °C. Before imaging, optimal antibody concentrations were determined. After removing the primary antibodies, the cells were washed three times for 5 minutes with PBS and incubated for 1 hour with secondary antibodies: Goat anti-mouse IgG Alexa Fluor TM 488 (A11029, Invitrogen, Massachusetts, USA) and Goat Anti-Rabbit IgG Secondary antibody Texas Red (T2767, Invitrogen, Massachusetts, USA).

Nuclei were visualized using DAPI Fluoromount-G® (Southern Biotech, Birmingham, USA). All incubations were conducted in a humidity chamber. Imaging was performed with a Leica DMi8 using a DC9000GT camera and a 63x oil immersion objective.

### Proximity ligation assay

HeLa cells were plated in a µ-Dish 35 mm, high Glass Bottom (Ibidi, Gräfelfing, Germany) at a density of 80,000 cells/dish. After fixation with 4 % paraformaldehyde for 10 minutes, cells were washed thrice with PBS and permeabilized using 0.5 % PBS-Triton-X for 10 minutes. Subsequently, the cells underwent three 5-minute washes with agitation using 0.05 % PBS-tween 20 and were blocked with Duolink® Blocking solution (Sigma-Aldrich, San Luis, USA) for 1 hour at 37 °C. Following blocking solution removal, a combination of anti-Ro52 and anti-Ro60 antibodies was incubated overnight at 4 °C in antibody diluent (Sigma-Aldrich, San Luis, USA). Cells were then washed thrice for 5 minutes each with 0.01 % PBS-tween with agitation. Subsequent steps involving ligation, and amplification were carried out using Duolink® *in situ* Detection reagents Red (Sigma-Aldrich, San Luis, USA). The ligase was incubated at 37 °C for 30 minutes and washed twice with 0.01 % PBS-tween with agitation. The polymerase was incubated for 100 minutes and washed successively with 1x and 0.01x SSC buffer for 5 minutes each. Nuclei were visualized using DAPI Fluoromount-G® (Southern Biotech, Birminghan, USA). All incubations at 37 °C were conducted in a humidity chamber. To induce calcium entry into the cells, either HCSS (120 mM NaCl; 0.8 mM MgCl_2_; 25 mM HEPES; 5.4 mM KCl; 30 mM Glucose; 2 mM CaCl_2_ pH7.4) alone or the ionophore A23187 (Sigma-Aldrich, San Luis, USA, C7522) was applied to the cells before fixation. Images were captured using a Leica DMi8 with a DC9000GT camera and a 63x oil immersion objective. Interactions were manually counted and expressed as the number of puncta per number of nuclei in each field.

### Cell cycle by flow cytometry

HeLa cells were plated in a 35 mm plate format, at a density of 80,000 cells/dish. After 24 hours, the cells were collected and transferred to a Flow cytometry tube. Cells were centrifuged for 5 min at 500 g and were fixed with 1 mL of 70 %, ice-cold EtOH for 1h at -20 ºC. Next, cells were centrifuged at 500 g for 5 min and stained in 700 µL of PI/RNASE Solution (Immunostep) for 24 h at 4 ºC. Fluorescence intensity was detected by flow cytometry with a BD FACSAria™ III cytometer equipped.

### Structure prediction

Ro52 is known to form a dimeric structure (Bateman et al., 2019[6], 2021[5]). Human Ro52 and Ro60 primary sequence data were obtained from UniProt (UniProt). Utilizing this information, the Ro52-Ro60 complex structure was predicted via the MDockPP Online Server (Burley et al., 2021[10]; Huang et al., 2013[22]; Huang and Zou, 2008[23], 2010[24]), starting from the primary sequences. This platform employs AlphaFold2 to construct monomeric structures (Jumper et al., 2021[25]), utilizing automatic template searches in the Protein Data Bank (PDB). The maximum template date chosen was the current date at that time (2023-09-14), without any constraints applied.

Subsequent refinement of the structure was conducted using the ColabFold relax_amber server (Mirdita et al., 2022[34]). Comprehensive assessments of structural integrity were performed through various tools provided by the SWISS-MODEL server, including MolProbity Stereochemistry, QMEANDisCo Model Quality Estimation (Chen et al., 2010[11]; Davis et al., 2004[14]; Studer et al., 2020[42]), along with utilities from the SAVES server such as ERRAT (Colovos and Yeates, 1993[13]), WHATCHECK (Hooft et al., 1996[21]), and PROCHECK (Laskowski et al., 1993[29]). Visualizing molecular interactions was enabled by LigPlot+ v.2.2.5 (EMBL-EBI; Laskowski and Swindells, 2011[30]; Wallace et al., 1995[44]), offering insights into the intricate interplays within the Ro52-Ro60 structure.

The estimation of protein-protein affinities was performed using the PDBePISA server v1.52 (EMBL-EBI; Krissinel and Henrick, 2007[28]; Shrake and Rupley, 1973[38]). Furthermore, the classification of protein-protein interactions relied on the HyPPI module within the ProteinsPlus server (Schneider et al., 2013[37]).

## Results and Discussion

### Cell-free experiments

**Figure 2:**
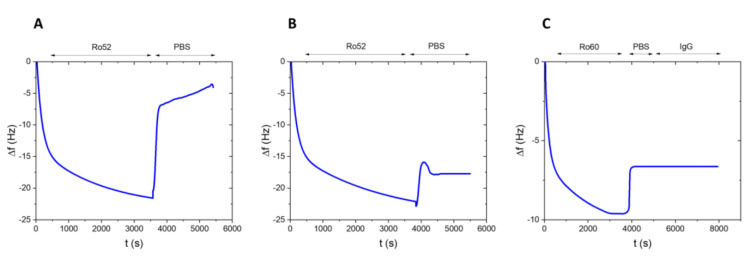
Monitoring signal for the interaction between Ro52 and Ro60-functionalized chips using QCM-D in two distinct solutions: (A) lacking CaCl_2, _(B) with a CaCl_2_ concentration of 100 mM in the solution. (C) QCM-D signal of the interaction between 12 U/mL IgG in solution and a Ro52-functionalized chip, pre-blocked using 50 mg/L Ro60 protein.

Utilizing QCM-D monitoring, we examined the kinetics of the Ro52-Ro60 interaction (Figure 2A(Fig. 2)). Chips functionalized with Ro60 were exposed to a solution containing Ro52 at a concentration of 300 mg/L. At that moment, the frequency rapidly decreased at a rate of -2.7 mHz/s, reaching -22.5 Hz and revealing the interaction between both proteins. After rinsing with 1x PBS, most of the initially attached protein was removed at a similar rate. However, the baseline was incompletely restored, leaving a minor fraction of Ro52 protein irreversibly bound, resulting in an 83 % decrease in the interaction signal and stabilizing around -4 Hz. Conversely, exposure of Ro60-functionalized chips to a solution containing bovine serum albumin at a concentration of 700 mg/L exhibited minimal interaction with the Ro60 substrate (|Δf| < 1 Hz), indicating the substrate's high specificity. These results highlight a reversible interaction between Ro52 and Ro60 proteins.

Figure 2B(Fig. 2) displays the QCM-D signal derived from a Ro60-functionalized chip upon injecting 50 mg/L Ro52 protein in a 100 mM calcium chloride solution. This setup elicited a rapid interaction rate with the Ro60 functionalized chip. Under these conditions, the interaction signal decreases significantly (-22 Hz, akin to the previous experiment albeit with a lower Ro52 protein concentration), and only a minute amount of Ro52 protein was removed post-rinsing with 1x PBS (approximately a 20 % decrease in the interaction signal, reaching a signal around -18 Hz). Consequently, the affinity between Ro52 and Ro60 proteins increases in these experimental conditions. The Ro60 protein exhibits a six-fold higher affinity for binding Ro52 in the presence of CaCl_2_ compared to a solution without this salt, underscoring the pivotal role of counterions in the Ro60-Ro52 interaction. This observation is consistent with the existence of a calcium-dependent MIDAS in the Ro60 structure.

Considering the data in Figure 2(Fig. 2), we estimated the association rate constant for Ro60-Ro52 complex formation using a one-to-one binding kinetics model. In the absence of CaCl_2_, the association kinetic constant for the Ro60-Ro52 interaction was 449 ± 16 M^-1^ s^-1^. However, in a 100 mM CaCl_2_ solution, it substantially increased to 2,600 ± 100 M^-1^ s^-1^, six times higher than the previous experimental conditions. This further confirms the regulatory role of CaCl_2_ in the Ro60-Ro52 interaction, mediated through Ro60's MIDAS. Moreover, the apparent equilibrium constant for the association between Ro60 and Ro52 proteins in a 100 mM CaCl_2_ solution, (3.7 ± 0.3) x 10^6^ M^-1^, was at least twenty-five times higher than the value estimated in the absence of CaCl_2_ in solution, (1.43 ± 0.08) x 10^5^ M^-1^. These observations highlight the critical involvement of the MIDAS motif in mediating these protein interactions.

In the cytoplasm, Ro52 functions as a robust Fc receptor, relying on its PRY-SPRY domain. This led to the conjecture that Ro60 might imitate these fragments, acting as a binding inhibitor that hinders Fc regions from engaging Ro52. To validate this idea, the Ro52 protein was immobilized onto a QCM-D chip. Subsequently, the chip was exposed to 50 mg/L Ro60 protein, and the interaction between this modified chip and a 12 U/mL IgG solution was monitored (Figure 2C(Fig. 2)). Surprisingly, the antibodies (around 150 kDa) failed to displace the Ro60 protein (60 kDa), resulting in minimal frequency changes (approximately 0 Hz). This suggests that the interaction between Ro52 and Ro60 proteins necessitates the involvement of the PRY-SPRY domain.

### Computational analysis

In line with our *in vitro* experiments, we conducted the computational analysis to uncover the structure of the Ro60-Ro52 complex. Using the hierarchical protein docking algorithm (MDockPP) based on the AlphaFold2 method, the structure of this complex was derived from its sequence. MDockPP has shown remarkable accuracy in predicting protein complex structures (Lensink et al., 2021[31]). Consequently, the initial evaluation of the deduced structure exhibited favorable scores across various parameters, including MolProbity (2.69), Clash (25.67), QMeanDisCo global score (0.76 ± 0.05), and ERRAT overall quality factor (89.6978) (Colovos and Yeates, 1993[13]; Hooft et al., 1996[21]; Studer et al., 2020[42]). Subsequent refinement using the ColabFold relax_amber server (Mirdita et al., 2022[34]) significantly enhanced the structural metrics (MolProbity 1.62, Clash 2.2, QMeanDisCo global score 0.77 ± 0.05, ERRAT overall quality factor 93.8676), indicating resolution of clashes within the complex.

**Figure 3:**
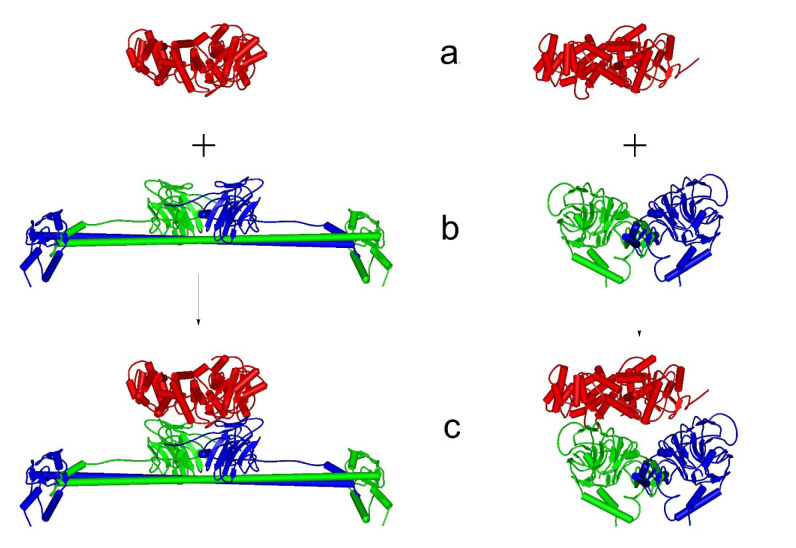
Structure of the Ro52-Ro60 complex derived from MDockPP and refined with the ColabFold relax_amber method, (a) represents the Ro60 protein, (b) shows the Ro52 homodimer and (c) displays the entire complex. Left, frontal view. Right, lateral view. In these depictions, Ro52 monomer chain A is highlighted in green, Ro52 monomer chain B in blue, and Ro60 in red. The corresponding pdf file is available in the Supplementary information.

In Figure 3(Fig. 3), the structure of the Ro60-Ro52 complex is depicted. In alignment with our experimental results, the toroid-shaped Ro60 protein is positioned within this complex nestled between the two PRY-SPRY domains of the Ro52 homodimer, resembling the structural configuration employed by Ro52 for antibody recognition (Foss et al., 2015[19]). Ro60 aligns horizontally with the homodimer's coiled-coil domain. Supplementary Information (SI) includes the pertinent PDB file and assessments from the SWISS-MODEL Structure Assessment and SAVES server.

**Figure 4:**
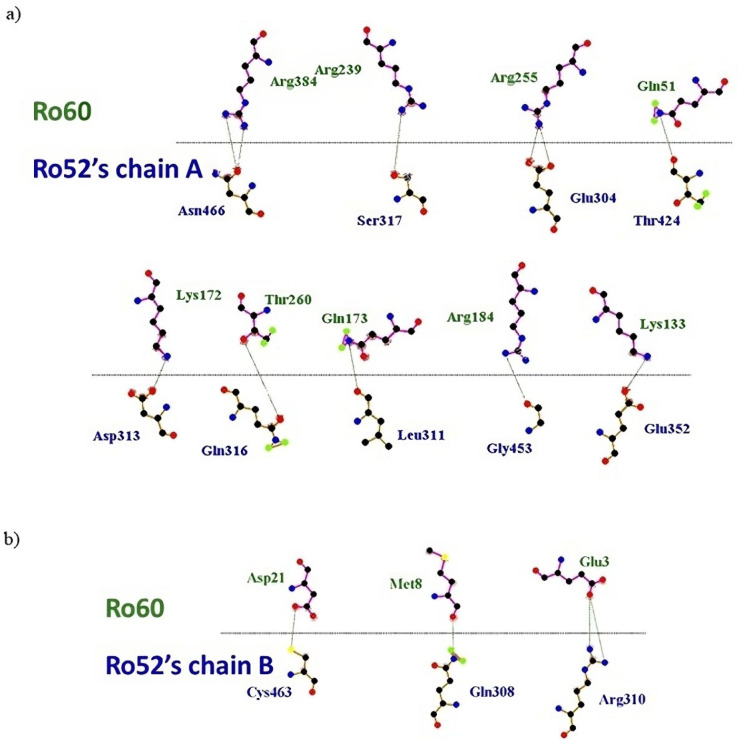
Schematic representation illustrating the hydrogen bond strong interactions between Ro60 (green) and Ro52 (blue) with their respective monomer chains: A (a) and B (b).

**Figure 5:**
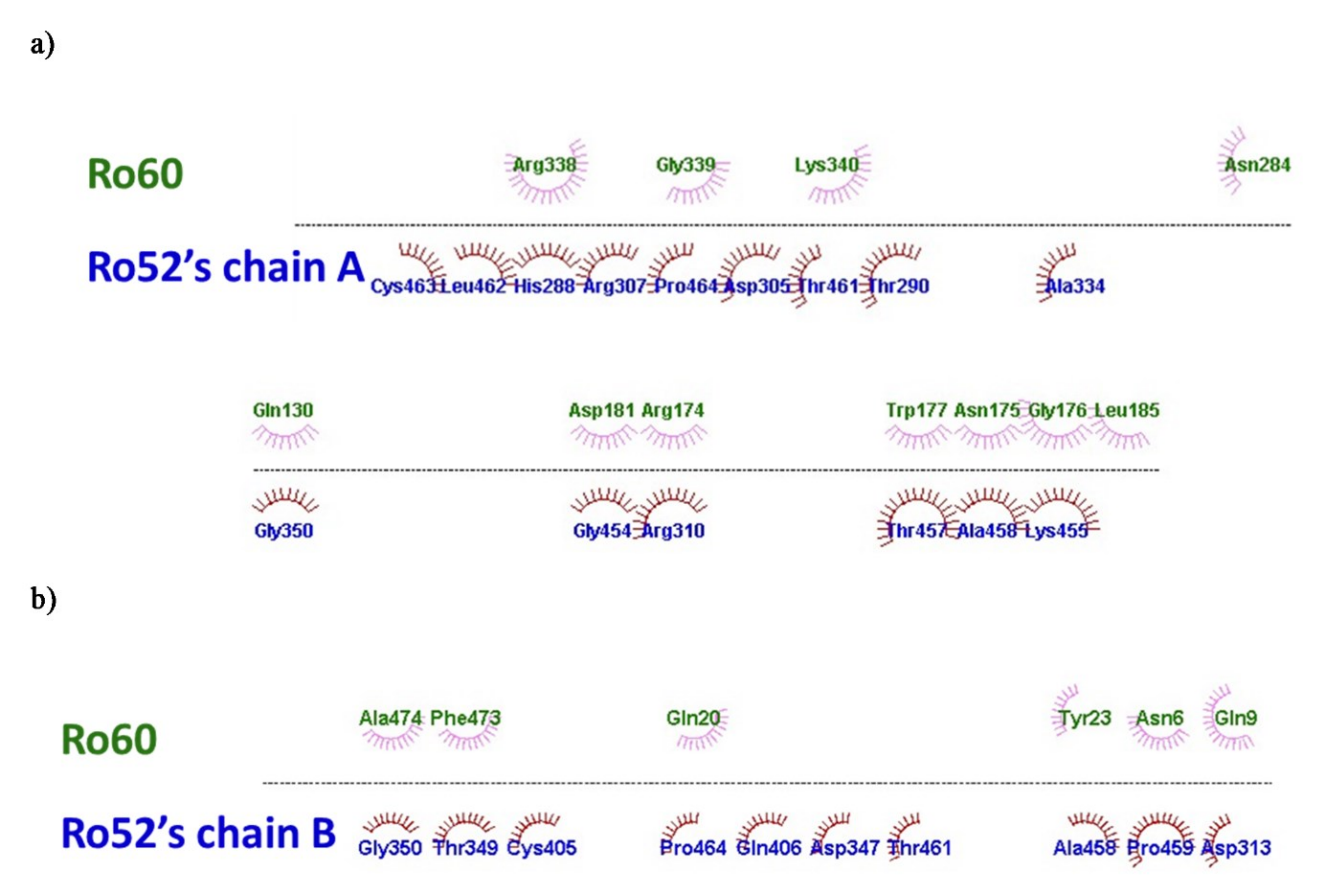
Schematic representation illustrating the hydrophobic and van der Waals weak interactions between Ro60 (green) and Ro52 (blue) with their respective monomer chains A (a) and B (b).

The LigPlot+ analysis uncovered intricate molecular interactions within the complex, showcasing distinct hydrogen bonding patterns (strong interaction, Figure 4(Fig. 4)) and delineating hydrophobic and van der Waals interactions (weak interactions, Figure 5(Fig. 5)). Ro52 monomer chain A exhibits robust interactions with several critical residues of Ro60, establishing essential hydrogen strong bonds. Specifically, residues R384, T260, R255, R239, R184, Q173, K172, K133, and Q51 of Ro60 predominantly engage in N-H^...^O type bonds with chain A. Remarkably, K133 plays a pivotal role in the Ro60-YRNA interaction, while a previous study identified residues R384, R255, and T260 in interactions of Ro60 with the Fc region of IgG (Juste-Dolz et al., 2019[26]).

On the other hand, the interaction between Ro52 homodimer chain B and Ro60 appears more limited, involving residues D21, M8, and E3 of Ro60. Notably, the bond between residue D21 of Ro60 and C463 of chain B forms an S-H^...^O type bond. This observation underscores the different involvement levels of Ro52 chain residues in interactions with Ro60.

Figure 5(Fig. 5) illustrates the limited hydrophobic weak interactions within the Ro60-Ro52 complex, primarily involving residue L185 from Ro52 chain A, along with residues A474 and F473 from chain B. The hydrophobic interactions are relatively stronger than other weak intermolecular forces. However, van der Waals interactions are more substantial in the Ro60-Ro52 complex. These interactions predominantly occur between Ro52 chain A and residues Q130, R174, N175, G176, W177, D181, L185, N284, K340, R338, and G339 of Ro60. Particularly, interactions involving residues N175, G176, W177, D181, and L185 significantly contribute to the Ro60-YRNA interaction (Stein et al., 2005[41]).

Experimentally, the presence of calcium ions significantly influences the Ro60-Ro52 interaction in solution. Our focus is specifically on examining the role of Ro60's MIDAS domain in this context. The MIDAS domain, spanning residues F371 to Q383 (FLLAVDVSASMNQ) (Clancy et al., 2022[12]), binds divalent cations, especially Ca^2+^, via S378 to S380. Hence, it notably affects the conformation of R384, located adjacent to the MIDAS sequence. This observation elucidates the connection between Ro60's affinity for Ro52 and the calcium ion concentration *in vitro*, as the position of R384 hinges on the conformation of the calcium ion-dependent site of Ro60. R384 within the Ro60-Ro52 complex forms a robust hydrogen bond with Ro52 residue N466 (refer to Figure 5(Fig. 5)). Earlier studies also highlighted the involvement of Ro60's residue R384 in interactions with the Fc region of IgG (Juste-Dolz et al., 2019[26]).

**Figure 6:**
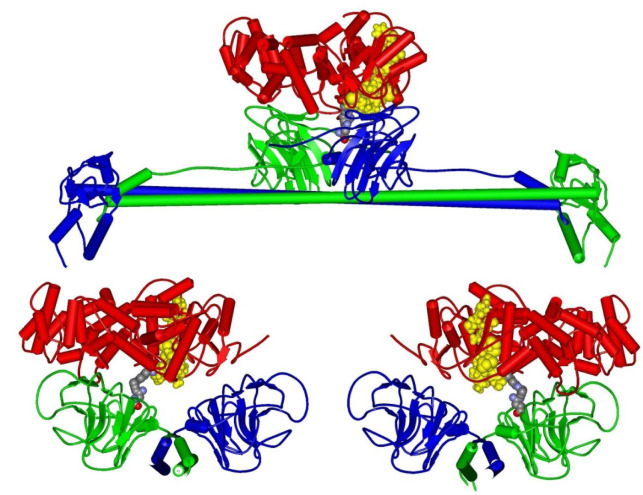
Three schematic views of the Ro60-Ro52 complex. Top: frontal perspective. Bottom: left and right profiles. Color legend: Red represents Ro60, Green is Ro52 chain A, Blue stands for Ro52 chain B, and Yellow denotes MIDAS. Interacting residues: Ro60-R384 and Ro52-N466S, shown in Corey-Pauling-Koltun atom display style.

Figure 6(Fig. 6) illustrates the positioning of the MIDAS domain within the Ro60-Ro52 complex, emphasizing its proximity to the protein-protein interface and pinpointing the interaction residues nearest to MIDAS. In this Figure, the importance of Ro60's residue R384 in shaping the Ro60-Ro52 complex is evident, owing to its strategic placement at the interface between the Ro60 protein and the A-chain of the Ro52 homodimer.

Despite these robust binding indications, closer examination revealed weak predicted free energies, potentially owing to steric repulsions. The detailed LigPlot+ software output is provided in the Supplementary information.

Utilizing the PDBePISA web server, estimated affinities between protein chains were calculated in terms of Δ^i^G, representing the solvation-free energy gain upon interface formation in kcal/mol. This metric is derived from the difference in solvation energies between isolated structures and the complex, with a negative Δ^i^G indicating a positive protein affinity. It is important to note that hydrogen bonds and electrostatic interactions at the interface do not influence this value (Krissinel and Henrick, 2007[28]). The analysis revealed significantly stronger interaction in the Ro52 monomers (-97.3 kcal/mol) compared to the Ro60-Ro52 complexes (1.8 kcal/mol for chain A, -1.2 kcal/mol for chain B) (Table 1(Tab. 1)).

The PDBePISA server incorporates P-statistics to gauge the uncertainty surrounding these energies. This statistical metric evaluates the probability of obtaining a Δ^i^G value lower than the observed one by randomly selecting interface atoms to match the reported interface area. It serves as an indicator of the unexpected nature of the interface's energy dynamics. For example, a P-value of 0.5 indicates an average Δ^i^G value within the given structures, reflecting predictable interface characteristics. A P-value exceeding 0.5 suggests potential crystal packing artifacts, pointing to a less hydrophobic interface than anticipated. Conversely, a P-value below 0.5 signifies unexpectedly high hydrophobicity, indicating potential variability in the interface surface due to the interaction's nature. A P-value of 0 signifies that no other interface within the observed area possesses a lower Δ^i^G, making the interface unique on the protein surface. The resulting P-values and other interface analysis outcomes are detailed in Table 1(Tab. 1).

Table 1(Tab. 1) shows how the interaction between the two Ro52 monomers is notably stronger than each monomer's interaction with Ro60. This is logical since it is a homodimer in solution. The P-values also reveal considerable uncertainty in the energies (Δ^i^G) of Ro60's interactions with the Ro52 monomers. This uncertainty suggests a weak interaction, indicating no permanent complex should form. As commented above, it is a reversible complex. Because of the relatively weak binding, the complex exists in dynamic equilibrium. Although the numerical value for the Ro60 interaction with the A-chain of Ro52 (1.8 kcal/mol) appears higher compared to the other interaction (-1.2 kcal/mol), the discrepancy lacks statistical significance due to the substantial uncertainty reflected in the associated P-values. However, it is evident that the Δ^i^G values for these interactions are small.

The determination of equilibrium constants for the solvation-free energy emphasizes the transient and dynamically stable nature of the Ro60-Ro52 complex. According to chemical thermodynamics, solvation-free energy correlates with the equilibrium constant through Δ^i^G = −RTln(^i^K), where Δ^i^G represents the solvation-free energy gain, R is the gas constant, T is the temperature, and ln(^i^K) stands for the natural logarithm of the solvation equilibrium constant. These equilibrium constants were calculated at 310 K. The values in Table 2(Tab. 2) validate the transient behavior of the Ro60-Ro52 complex.

To validate the reversibility of the complex, the HyPPI algorithm enabled the classification of the Ro60-Ro52 complex as either permanent, transient, or crystalline artifacts. Permanent complexes remain stable when combined but would denature when dissociated, while transient complexes feature dynamic association and dissociation at equilibrium in solution. Crystalline artifacts lack biological function, forming artificially during crystallization. For the Ro60 and Ro52 interface, probabilities were assigned: 1 % for a permanent complex, 96 % for a transient one, and 3 % for a crystalline artifact. This classification supports the PDBePISA results, further emphasizing the complex's transient nature. Moreover, it corroborates the previously estimated K_a_ = (3.7 ± 0.3) x 10^6^ M^-1^, indicative of a weak transient complex (Acuner Ozbabacan et al., 2011[1]).

Such transient complexes, varying in affinity and duration, are crucial for diverse biological processes such as biochemical pathways and singling cascades in the cell, and classified based on lifetime or stability (Acuner Ozbabacan et al., 2011[1]). These interactions facilitate rapid cellular responses to external stimuli (Acuner Ozbabacan et al., 2011[1]). Ro52 protein's involvement in intercellular viral particle proteolysis could align seamlessly with the role of transient complexes in these crucial cellular processes.

### In vitro cellular experiments

While the methodologies used serve as fundamental tools for decoding protein-protein interactions, their predictions might not consistently mirror the dynamics observed in living cells. To confirm our earlier observations, we commenced an *in vitro* investigation into the Ro52-Ro60 interaction in HeLa cells. We first performed an immunostaining of Ro52 and Ro60 in HeLa cells in order to visualize the proteins and titrate the antibodies.

**Figure 7:**
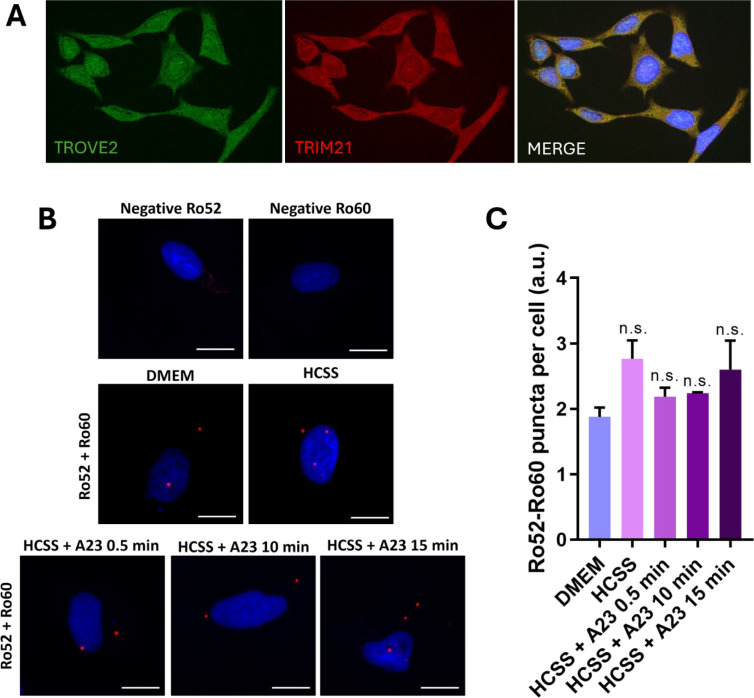
Assessment of Ro52-Ro60 interaction in HeLa cells. (A) Representative microscopy images illustrating the localization of Ro60 (TROVE2) and Ro52 (TRIM21) in HeLa cells through immunostaining assays. The right panel shows the merge image of anti-Ro60 (green) and anti-Ro52 (red). Co-localized proteins appear yellow in the overlaid images. Nuclei were stained with DAPI (blue). (B) Representative microscopy images of Proximity Ligation Assay evaluating the interaction of Ro52-Ro60 in different conditions. Cells were incubated with culture medium (DMEM) or HCSS for 30 min. Besides, HCSS was incubated alone or with A23187 (A23) for 0.5, 10, and 15 min to stimulate Ca^2+^ entry to the cell. Scale Bar: 30 µM. (C) Number of interactions (puncta) per cell in each condition. Three independent experiments were conducted for each condition (n=3), with a minimum of 300 cells analyzed for each experiment. Results are represented as mean±SEM. ANOVA significance: n.s. (non-significant)

The indirect immunostaining assays shown in Figure 7A(Fig. 7) (right and middle panels) revealed a predominant cytoplasmic expression of Ro60 and Ro52 proteins, consistent with previous literature (Boccitto and Wolin, 2019[8]; Arase et al., 2022[4]), alongside a minor presence within the cell nucleus. Moreover, the co-localization of Ro60 and Ro52 proteins can be observed within the cytoplasm (Figure 7A(Fig. 7), right panel).

Next, we proceeded with PLA to evaluate the interaction between Ro52 and Ro60 in HeLa cells. As a result, red-fluorescent dots representing the Ro60-Ro52 interaction were visible in the cytoplasm when PLA was performed in DMEM medium (Figures 7B and 7C(Fig. 7)). The count was 1.80 ± 0.15 puncta/cell compared to the Ro60 or Ro52 antibodies used as negative controls (0.3 ± 0.3 puncta/ field), confirming the interaction between the two proteins. Similar results were obtained when the cells we incubated with HCSS buffer (calcium-rich buffer, 2.7 ± 0.3 puncta/ cell). Therefore, this result, along with the co-localization of both proteins, suggests the formation of the Ro60-Ro52 complex in the cytoplasm of HeLa living cells. The limited number of detected interactions could reflect the presence of a weak transient complex, consistent with the observations reported in our initial experiments. It is important to note that the studied HeLa cells are primarily in the G_0_ and G_1_ phases of the cell cycle, with 57.5±0.9 % of the cells in these phases (see Figure S20 in the Supplementary information for details).

Finally, to elucidate the role of calcium ions in the Ro60-Ro52 interaction, PLA was conducted in live cells incubated with HCSS and stimulated for calcium entry using ionophore A23187. Despite this ionophore is commonly used to increase intracellular Ca^2+^ levels in intact cells, the number of complexes observed did not increase significantly under any of the tested conditions (Figure 7C(Fig. 7)). This suggests that calcium ions might not directly influence this interaction. As previously mentioned, this binding might be more impacted by the conformational changes in Ro60's residue R384.

## Conclusion

The QCM-D analysis unveiled a reversible bond between Ro52's homodimer and Ro60, showcasing a K_a_ = (3.7 ± 0.3) x 10^6^ M^-1^. The computational framework used not only provided structural insights but also shed light on the stability and intermolecular interactions, underscoring its biological relevance. Notably, the Ro60-Ro52 interface appeared transient with residue R384, situated near the MIDAS, influencing their binding dynamics. Validation within living cells via IIF and PLA showed the formation of the Ro60-Ro52 complex within HeLa cell cytoplasm.

The Ro60-Ro52 complex is a weak transient complex, with Ro60 found horizontally within the PRY-SPRY domains of the Ro52's homodimer. These domains play a critical role in recognizing intercellular antibodies, essential for the cellular self-defense mechanism. Crucial residues, notably K133, N175, G176, W177, D181, or L185 of Ro60, involved in the Ro60-Ro52 interaction, also participate in binding misfolded noncoding RNAs with Ro60, contributing to the quality control pathway for ncRNAs.

Our results highlight the presence of Ro60-Ro52 transient complex in living cells. Further exploration in various cellular contexts of the physiological role of these complexes is urgently needed. Understanding the roles of this weak transient complex in regulation of biochemical pathways is pivotal, potentially shaping a new understanding of the balance between the cellular self-defense system and the multifaceted functions of YRNA. These functions span various cellular processes such as DNA replication, RNA quality control, and cellular stress responses. This would help in the development of targeted therapies. Modifying the formation of the Ro60-Ro52 complex might emerge as a prospective therapeutic target for systemic autoimmune diseases, particularly considering the involvement of anti-SSA/Ro autoantibodies in these diseases.

## Notes

Pilar González-Cabo and David Gimenez-Romero (Department of Physical Chemistry, Faculty of Chemistry, University of Valencia, C/ Doctor Moliner 50, 46100, Burjassot, Spain; E-mail: giroda@uv.es) contributed equally as corresponding author.

## Declaration

### Supplementary information 

Selected results from the ERRAT, PROCHECK and SWISS-MODEL Structure Assessment servers. The PDB file of structure coordinates of the refined Ro60-Ro52 complex as well as the complete output of the SWISS-MODEL Structure Assessment, ERRAT, WHATCHECK, PROCHECK, and PDBePISA servers are available from the authors on request. Analysis of the cell cycle in HeLa cells by Flow Cytometry is also available.

### Acknowledgments

J. V. de J.-O. acknowledges Generalitat Valenciana, Conselleria de Innovación, Universidades, Ciencia y Sociedad Digital, Dirección General de Ciencia e Investigación, grants to emerging research groups CIGE/ 2022/59, Spain, for financial support.

### Conflict of interest

The authors declare that they have no conflict of interest.

## Supplementary Material

Supplementary information

## Figures and Tables

**Table 1:**
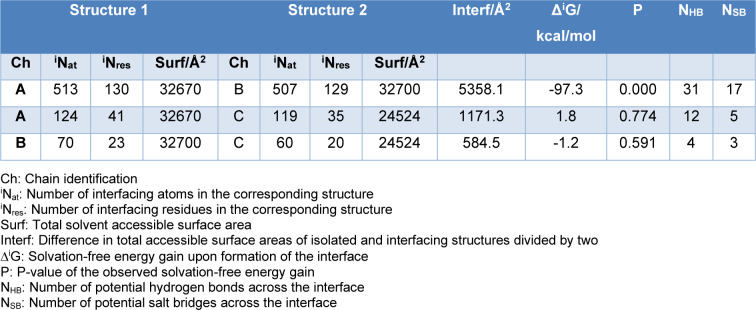
Interface analysis by using the PDBePISA server, where 'A' denotes the A-chain of Ro52, 'B' represents the B-chain, and 'C' corresponds to Ro60.

**Table 2:**
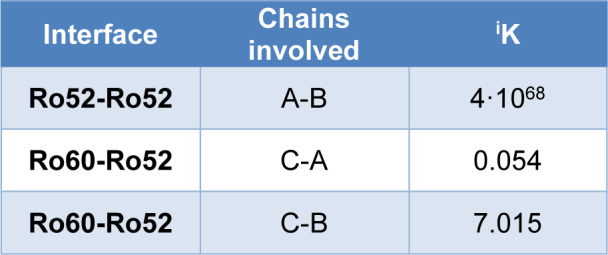
Solvation equilibrium constants for each pair of chains in the complex, where 'A' denotes the A-chain of Ro52, 'B' represents the B-chain, and 'C' corresponds to Ro60.

**Figure 1:**
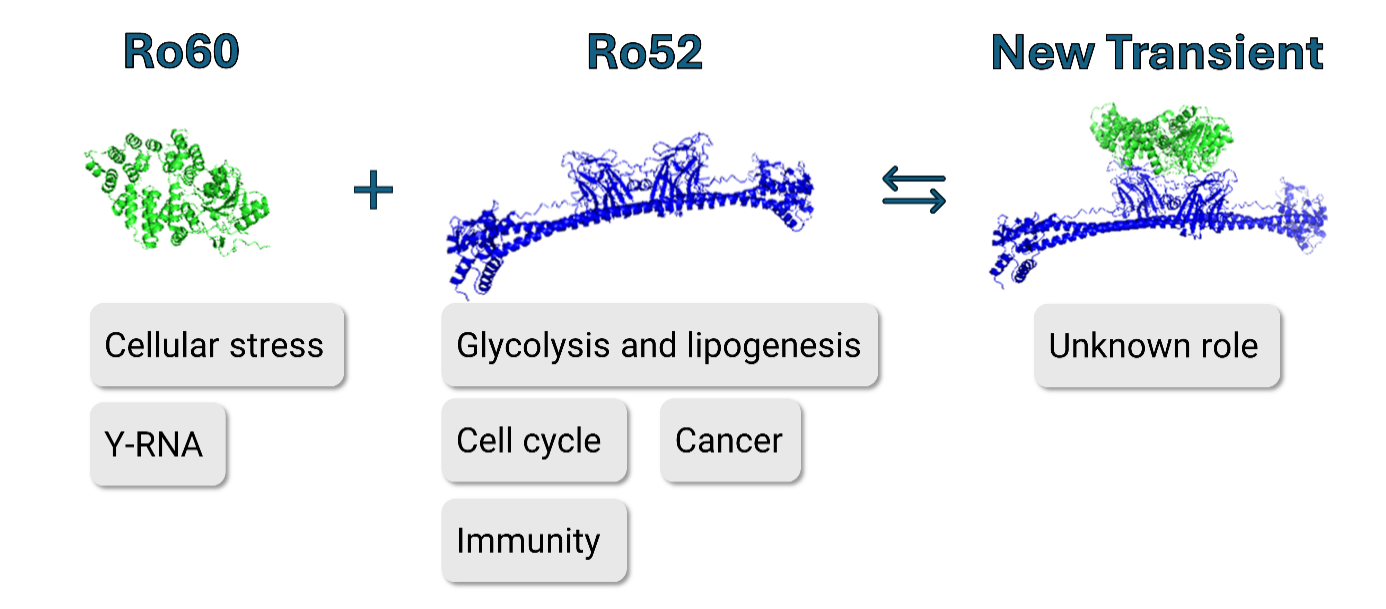
Graphical abstract
