# Supplementary material for: Unveiling the Ro60-Ro52 complex
Source: EXCLI J. 2024 Jun 10;23:888–903. doi: 10.17179/excli2024-7141 (PMC11231564; doi:10.17179/excli2024-7141)
Supplement: Supplementary information [file EXCLI-23-888-s-001.pdf]

EXCLI Journal 2024;23:888-903 – ISSN 1611-2156
Received: January 24, 2024, accepted: April 26, 2024, published: June 10, 2024

Supplementary information to:

Original article

UNVEILING THE Ro60-Ro52 COMPLEX

Laura R. Rodríguez[1,2,3], Jesus Vicente de Julián-Ortiz[4], Fernando Rubio de la Rúa[5], Augusto Juste-Dolz[6], Ángel Maquieira[7,8], Haydar A. Mohammad-Salim[4,9], Sofiane Benmetir[4,10], Federico V. Pallardó[1,2], Pilar González-Cabo[1,2*], David Gimenez-Romero[5*]

1 Department of Physiology, Faculty of Medicine and Dentistry. University of Valencia-INCLIVA, 46010 Valencia, Spain
2 CIBER Rare Diseases (CIBERER), 46010 Valencia, Spain
3 Present address: Stem Cells, Aging and Neurodegeneration Group, Department of Experimental Medical Science, Faculty of Medicine, Lund Stem Cell Center, Lund University, 22184, Lund, Sweden
4 Molecular Topology and Drug Design Research Unit, Department of Physical Chemistry, Faculty of Pharmacy, University of Valencia, Av. Vicent Andrés Estellés s/n, 46100 Valencia, Spain
5 Department of Physical Chemistry, Faculty of Chemistry, University of Valencia, C/ Doctor Moliner 50, 46100, Burjassot, Spain
6 Center for Research and Innovation on Bioengineering (Ci2B), Universitat Politècnica de València, Camino de Vera s/n, 46022 Valencia, Spain
7 Departamento de Química, Polytechnic University of Valencia, Camino de Vera s/n 46022, Valencia, Spain
8 Instituto Interuniversitario de Investigación de Reconocimiento Molecular y Desarrollo Tecnológico (IDM), Universitat Politècnica de València, Universitat de València, Camino de Vera s/n, 46022 Valencia, Spain
9 Faculty of Science, Department of Chemistry, University of Zakho, Zakho, Duhok 42001, Kurdistan Region, Iraq
10 Process and Environmental Engineering Laboratory (LIPE), Faculty of Chemistry, University of Science and Technology of Oran Mohamed BOUDIAF, P.O. Box 1503, El Mnaouer, 31000 Oran, Algeria

* **Corresponding authors:** Pilar González-Cabo, Department of Physiology, Faculty of Medicine and Dentistry. University of Valencia-INCLIVA, 46010 Valencia, Spain. E-mail: Pilar.Gonzalez-Cabo@uv.es
David Gimenez-Romero, Department of Physical Chemistry, Faculty of Chemistry, University of Valencia, C/ Doctor Moliner 50, 46100, Burjassot, Spain. E-mail: giroda@uv.es

https://dx.doi.org/10.17179/excli2024-7141

This is an Open Access article distributed under the terms of the Creative Commons Attribution License (http://creativecommons.org/licenses/by/4.0/).

EXCLI Journal 2024;23:888-903 – ISSN 1611-2156
Received: January 24, 2024, accepted: April 26, 2024, published: June 10, 2024

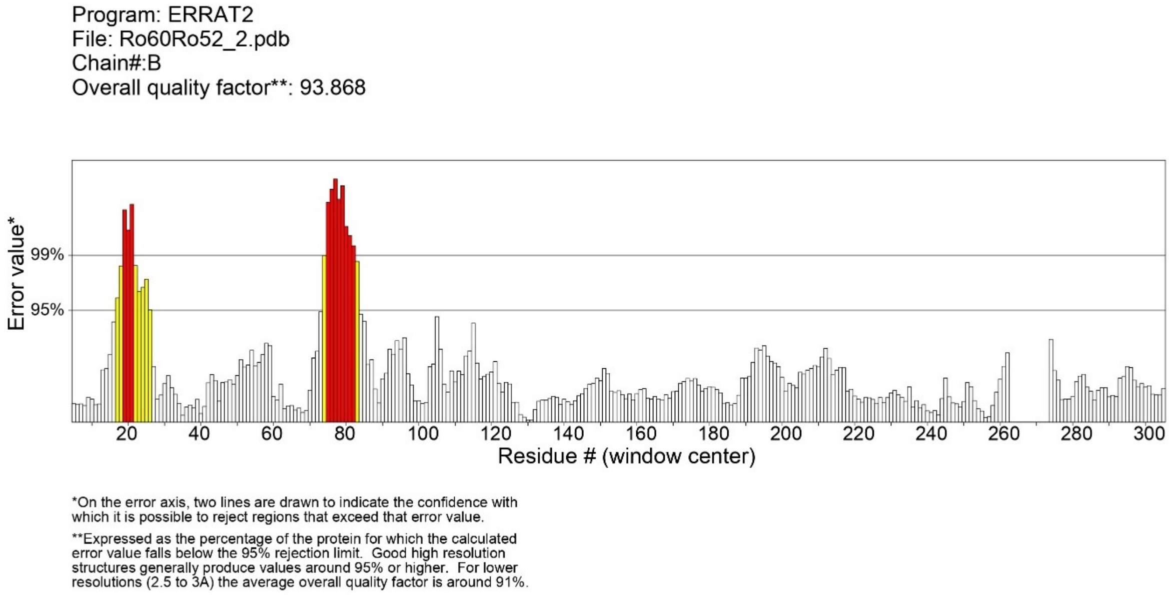

Figure S1: ERRAT2 values for the first 300 residues of the B-chain of Ro52

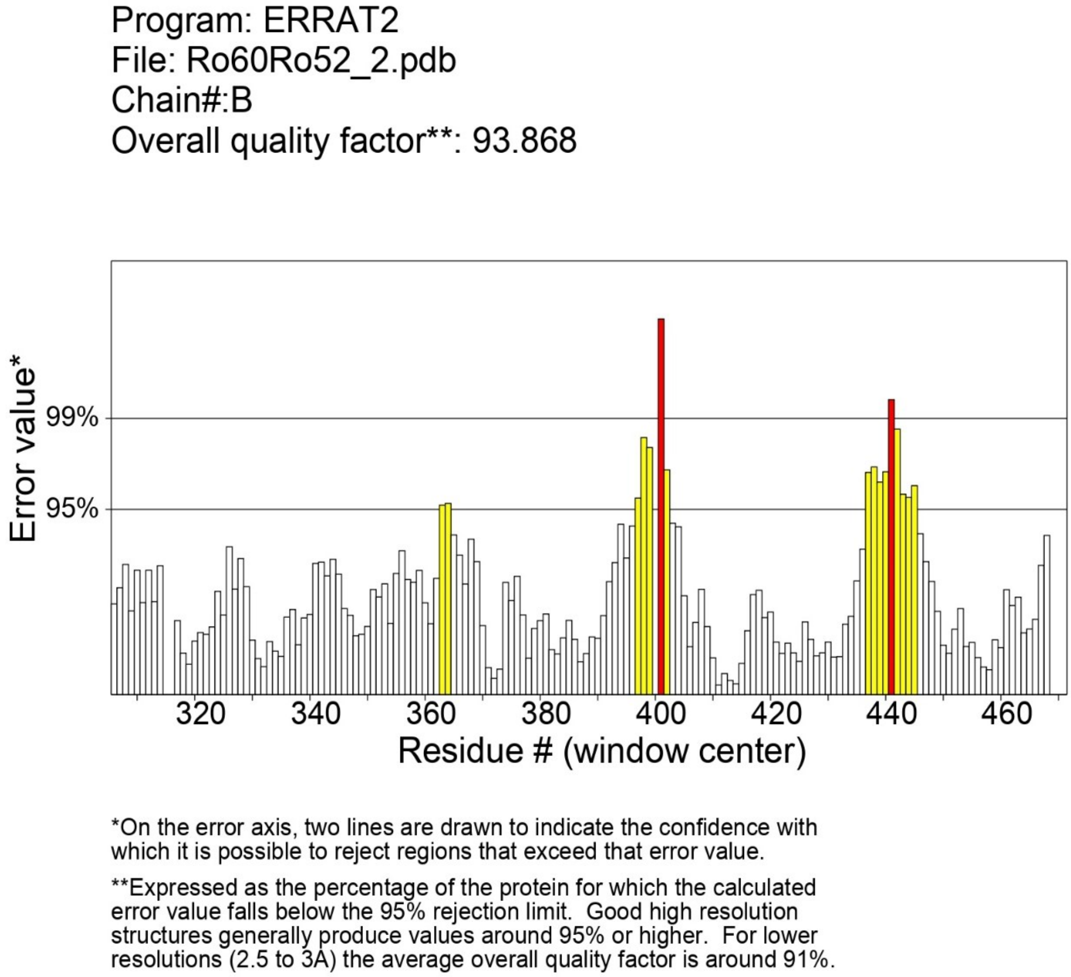

Figure S2: ERRAT2 values for residues 301 to the last residue of the B-chain of Ro52

EXCLI Journal 2024;23:888-903 – ISSN 1611-2156
Received: January 24, 2024, accepted: April 26, 2024, published: June 10, 2024

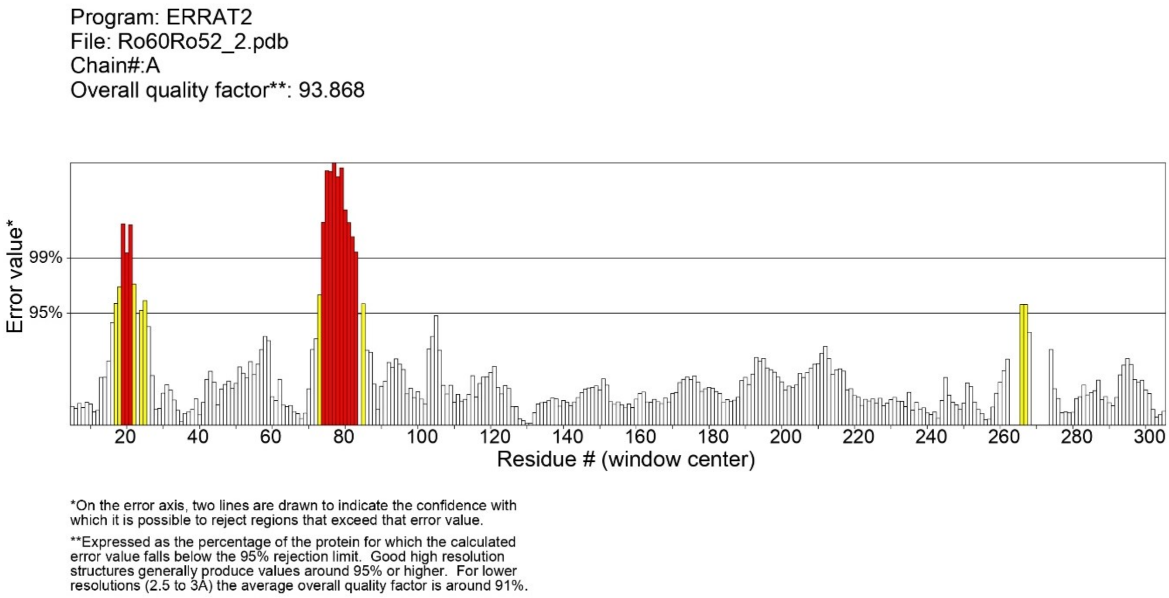

Figure S3: ERRAT2 values for the first 300 residues of the A-chain of Ro52

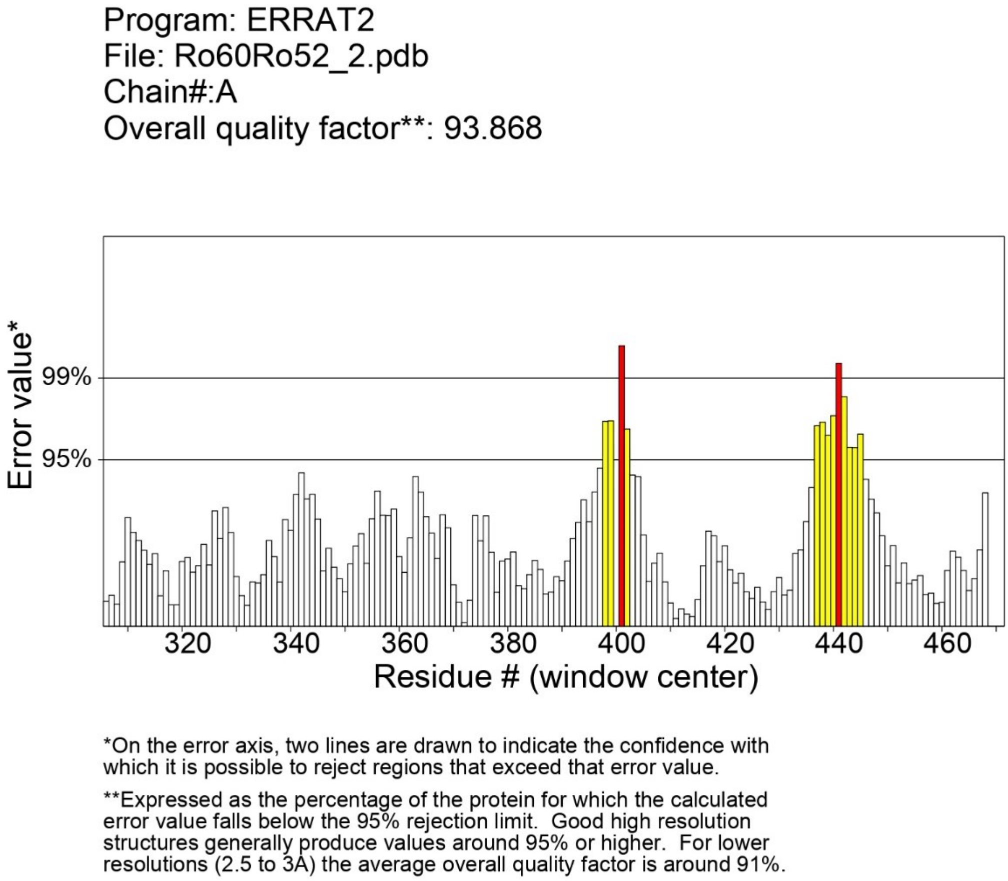

Figure S4: ERRAT2 values for residues 301 to the last residue of the A-chain of Ro52

EXCLI Journal 2024;23:888-903 – ISSN 1611-2156
Received: January 24, 2024, accepted: April 26, 2024, published: June 10, 2024

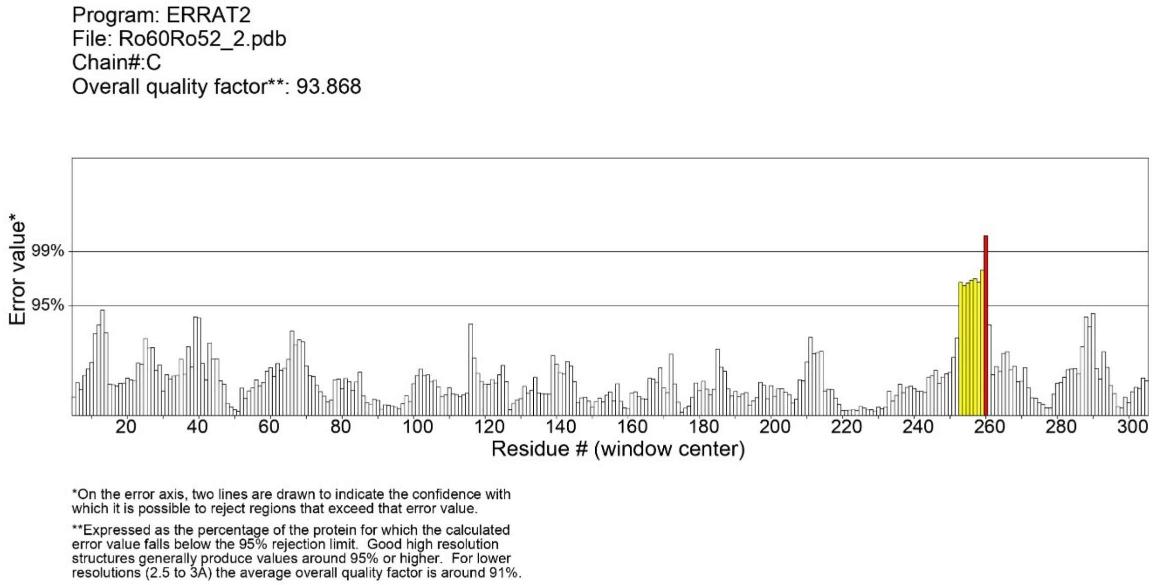

Figure S5: ERRAT2 values for the first 300 residues of Ro60

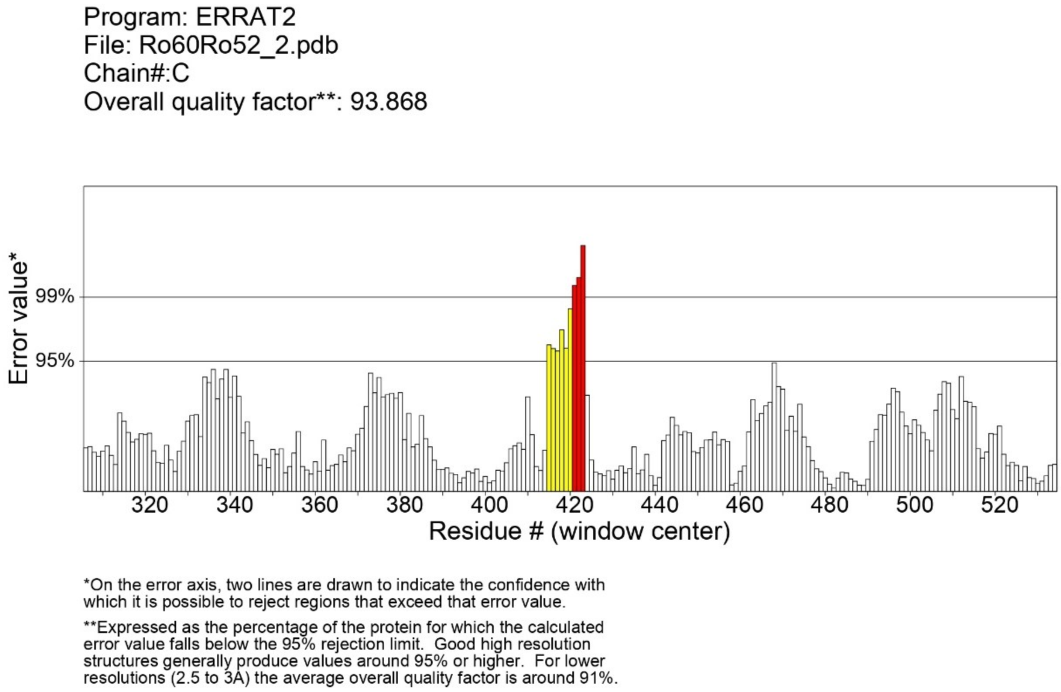

Figure S6: ERRAT2 values for residues 301 to the last residue of Ro60

EXCLI Journal 2024;23:888-903 – ISSN 1611-2156
Received: January 24, 2024, accepted: April 26, 2024, published: June 10, 2024

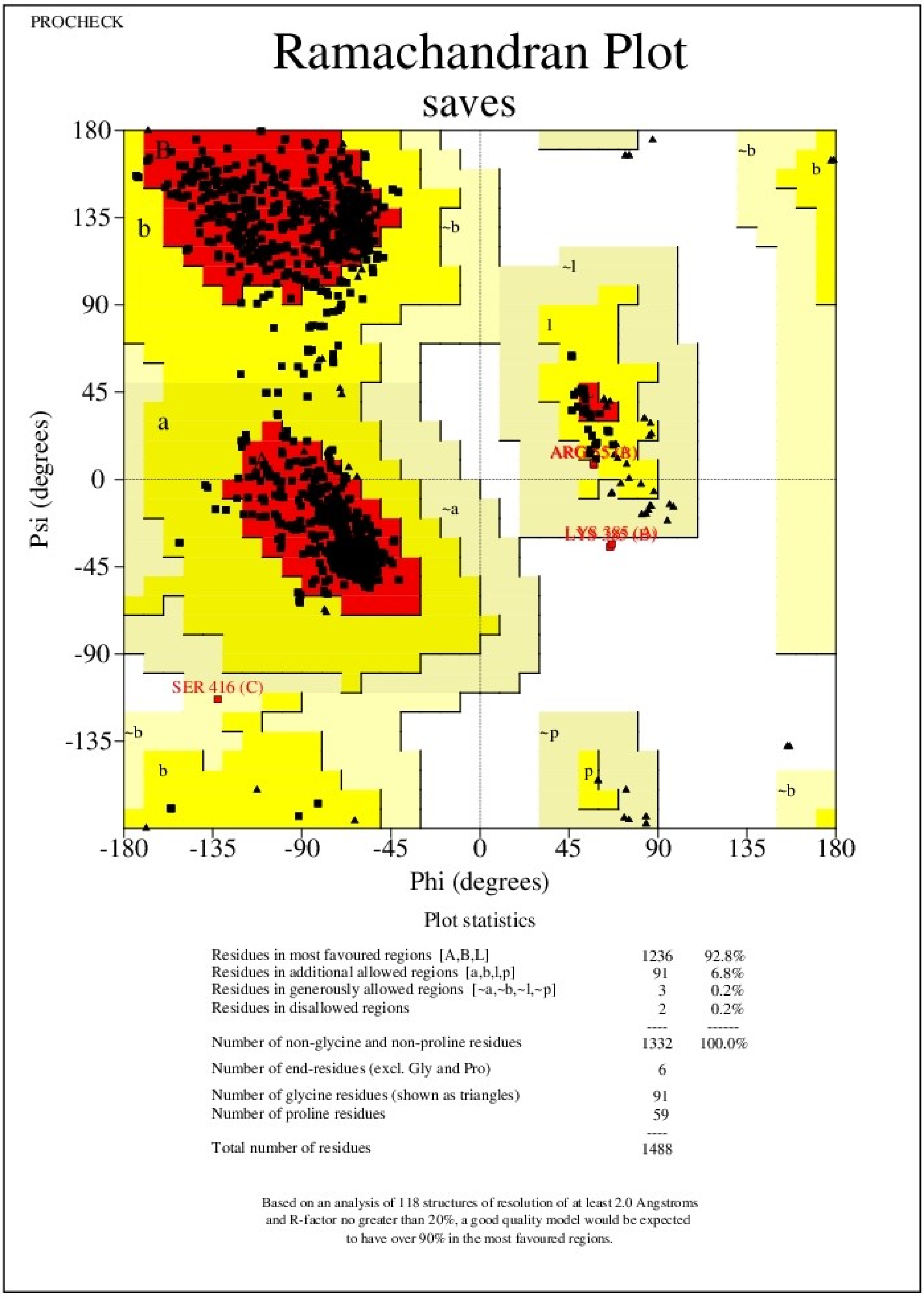

saves_01.ps

Figure S7: Ramachandran plot and statistics for the Ro60-Ro52 complex obtained with PROCHECK

Received: January 24, 2024, accepted: April 26, 2024, published: June 10, 2024

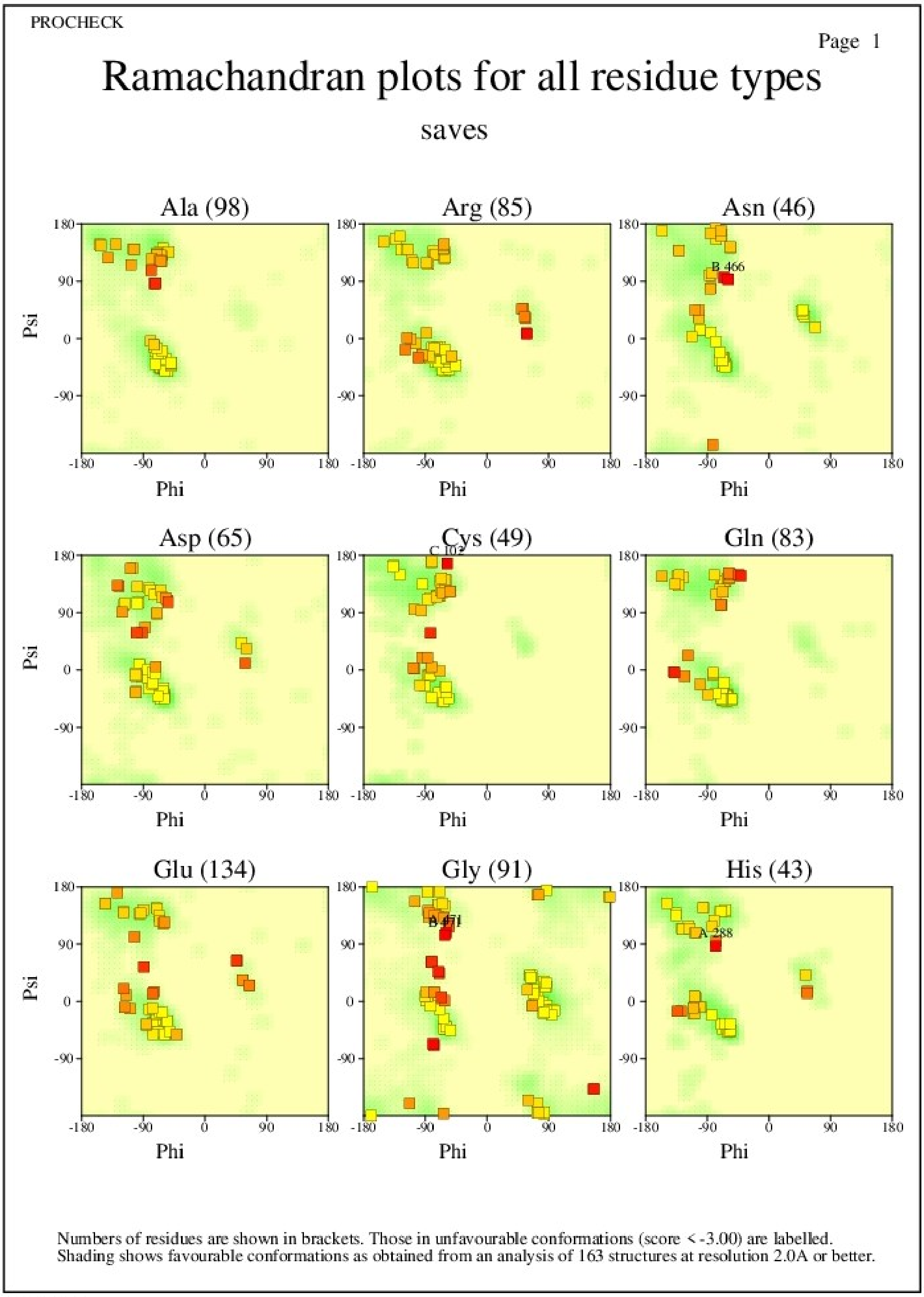

saves_02.ps

Figure S8: Ramachandran plot for the Alanine to Histidine residues of the Ro60-Ro52 complex obtained with PROCHECK

EXCLI Journal 2024;23:888-903 – ISSN 1611-2156
Received: January 24, 2024, accepted: April 26, 2024, published: June 10, 2024

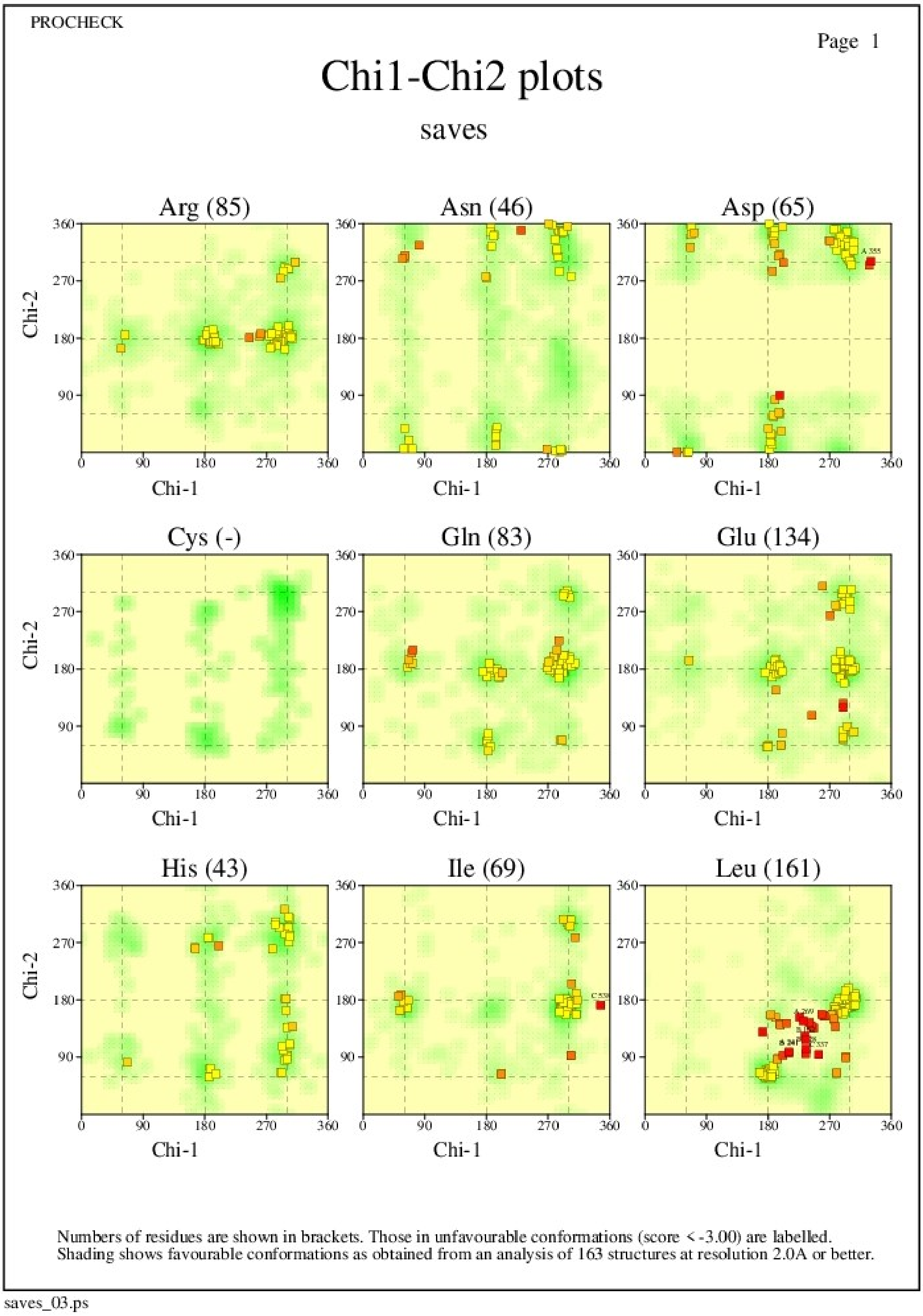

Figure S9: X_1 – X_2 plots of some residues of the Ro60-Ro52 complex obtained with PROCHECK

EXCLI Journal 2024;23:888-903 – ISSN 1611-2156
Received: January 24, 2024, accepted: April 26, 2024, published: June 10, 2024

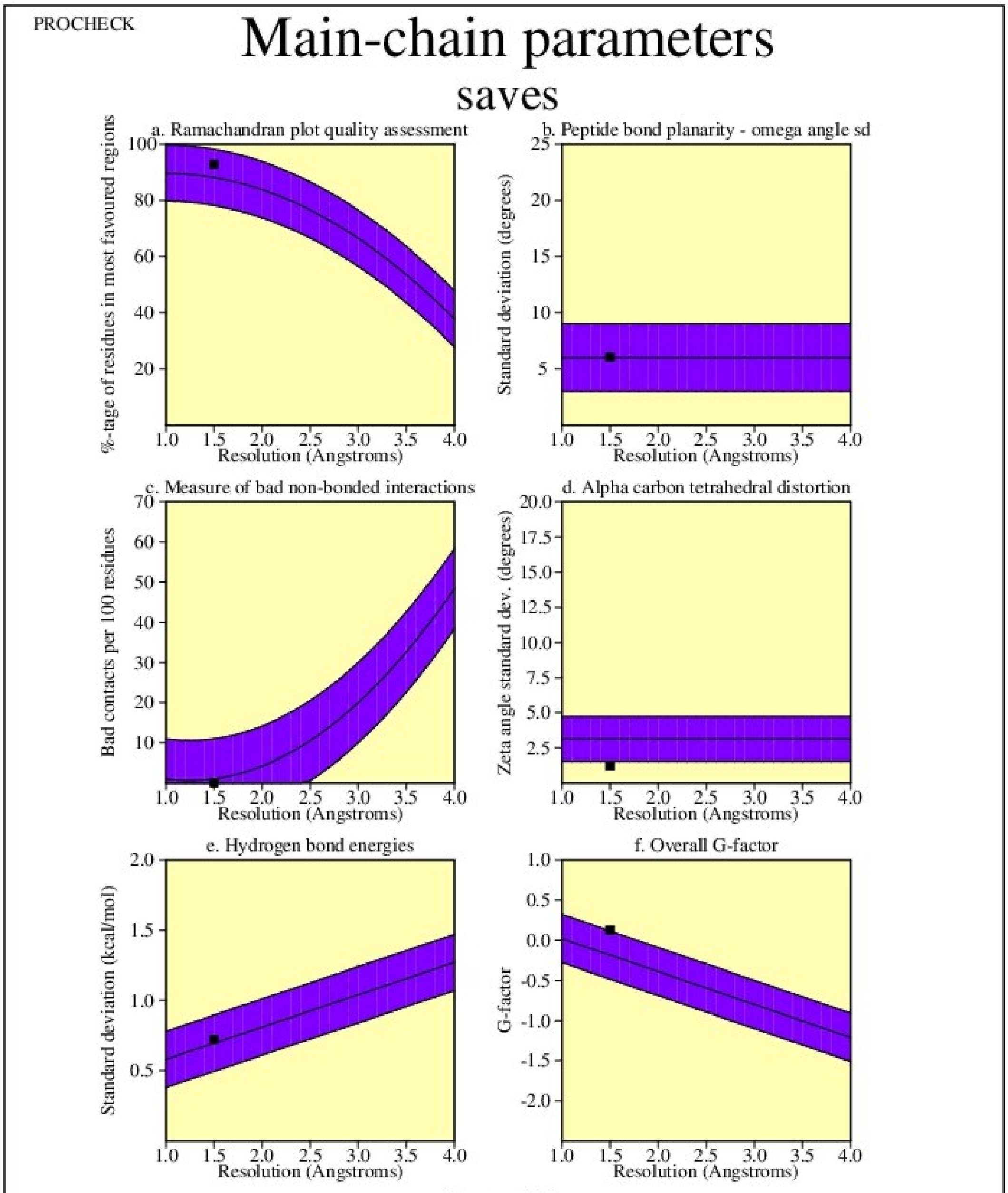

Plot statistics

Stereochemical parameter	No. of data pts	Parameter value	Comparison values Typical value	Band width	No. of band widths from mean	
a. %-tage residues in A, B, L	1332	92.8	88.2	10.0	0.5	Inside
b. Omega angle st dev	1483	6.1	6.0	3.0	0.0	Inside
c. Bad contacts / 100 residues	0	0.0	1.0	10.0	-0.1	Inside
d. Zeta angle st dev	1397	1.2	3.1	1.6	-1.2	BETTER
e. H-bond energy st dev	1004	0.7	0.7	0.2	0.1	Inside
f. Overall G-factor	1488	0.1	-0.2	0.3	1.0	BETTER

saves_04.ps

Figure S10: Parameters and statistics of the main chain of the Ro60-Ro52 complex obtained with PROCHECK

EXCLI Journal 2024;23:888-903 – ISSN 1611-2156
Received: January 24, 2024, accepted: April 26, 2024, published: June 10, 2024

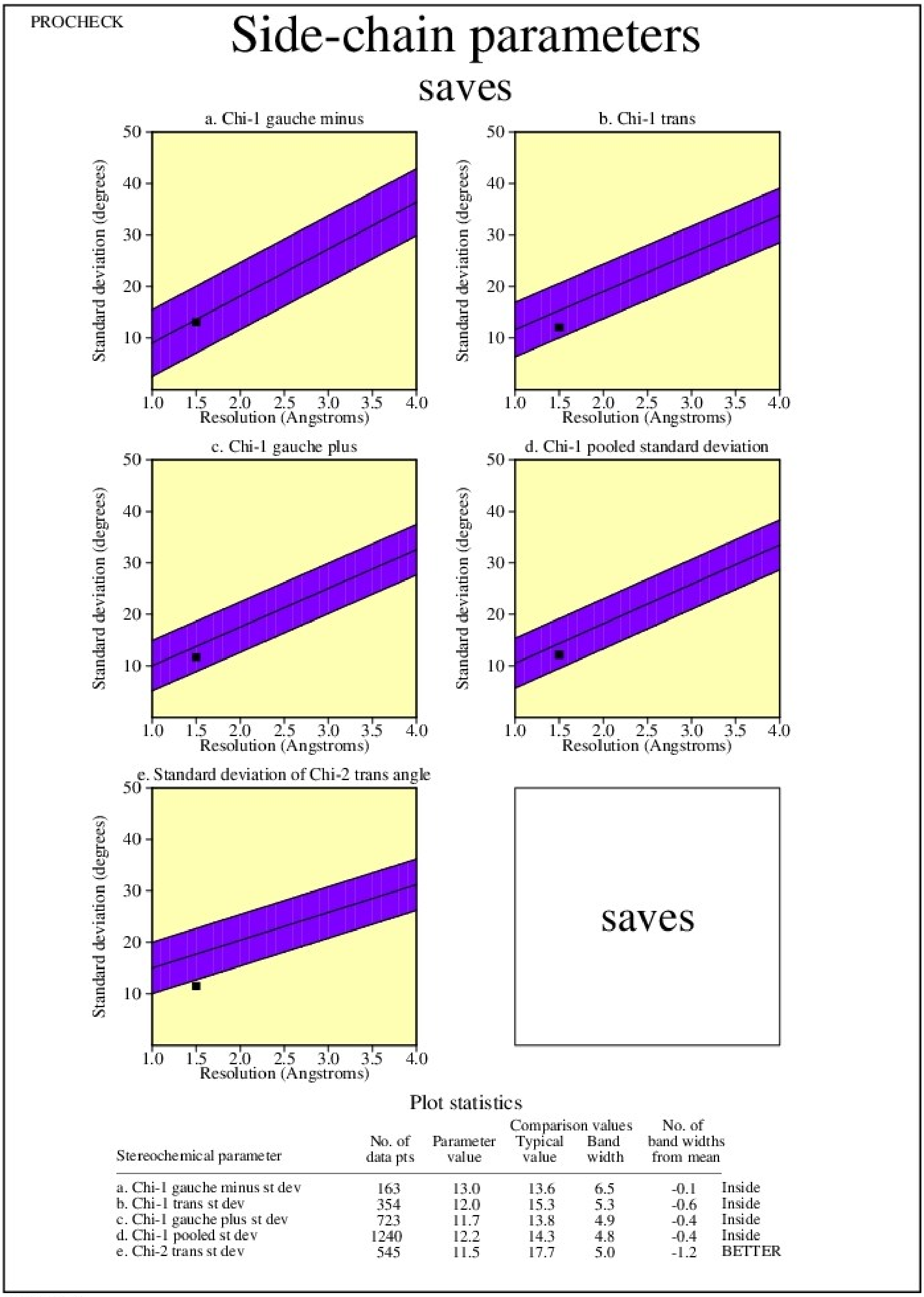

saves_05.ps

Figure S11: Parameters and statistics of the side chain of the Ro60-Ro52 complex obtained with PROCHECK

EXCLI Journal 2024;23:888-903 – ISSN 1611-2156
Received: January 24, 2024, accepted: April 26, 2024, published: June 10, 2024

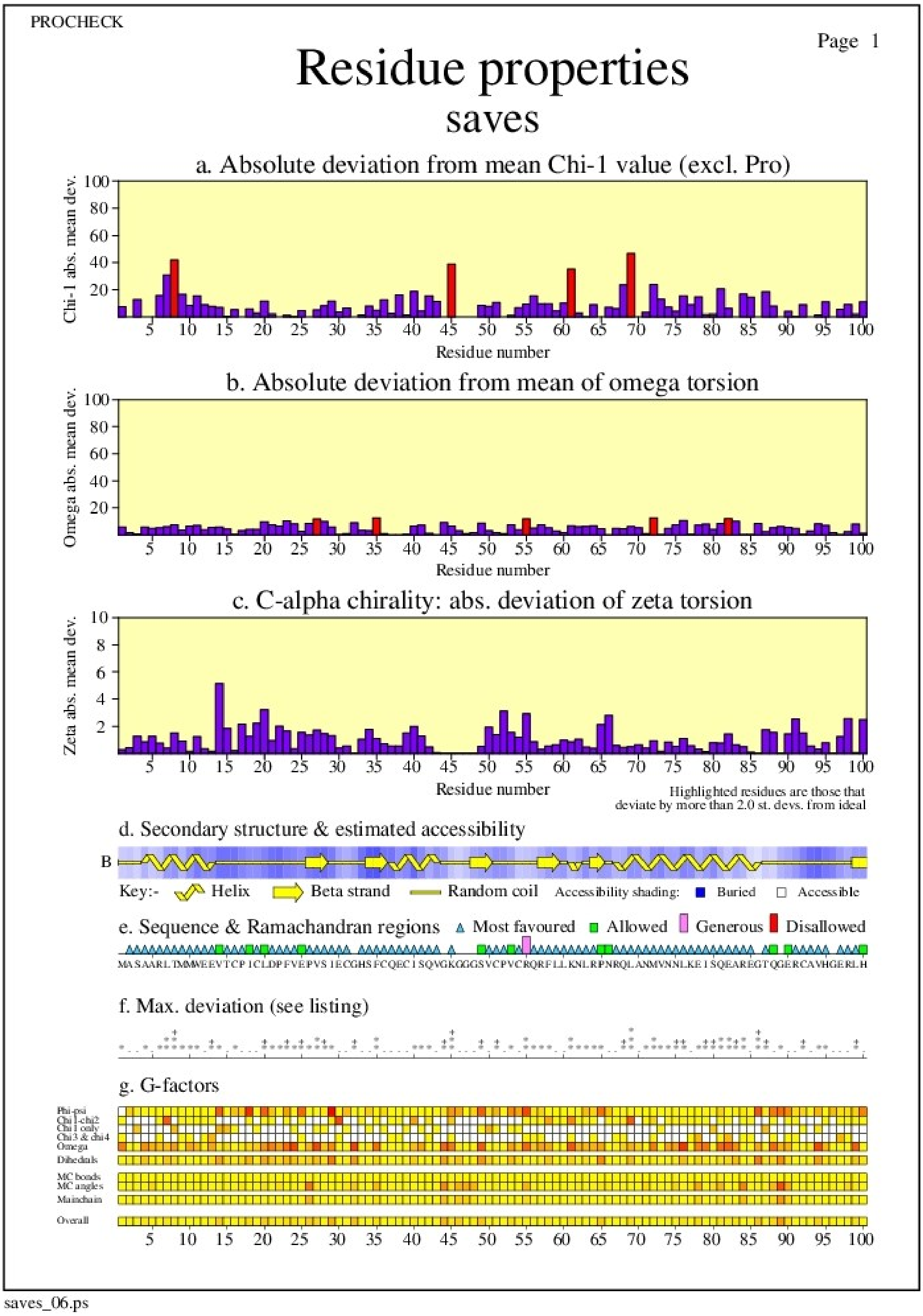

Figure S12: Properties of the residues of the Ro60-Ro52 complex obtained with PROCHECK

EXCLI Journal 2024;23:888-903 – ISSN 1611-2156
Received: January 24, 2024, accepted: April 26, 2024, published: June 10, 2024

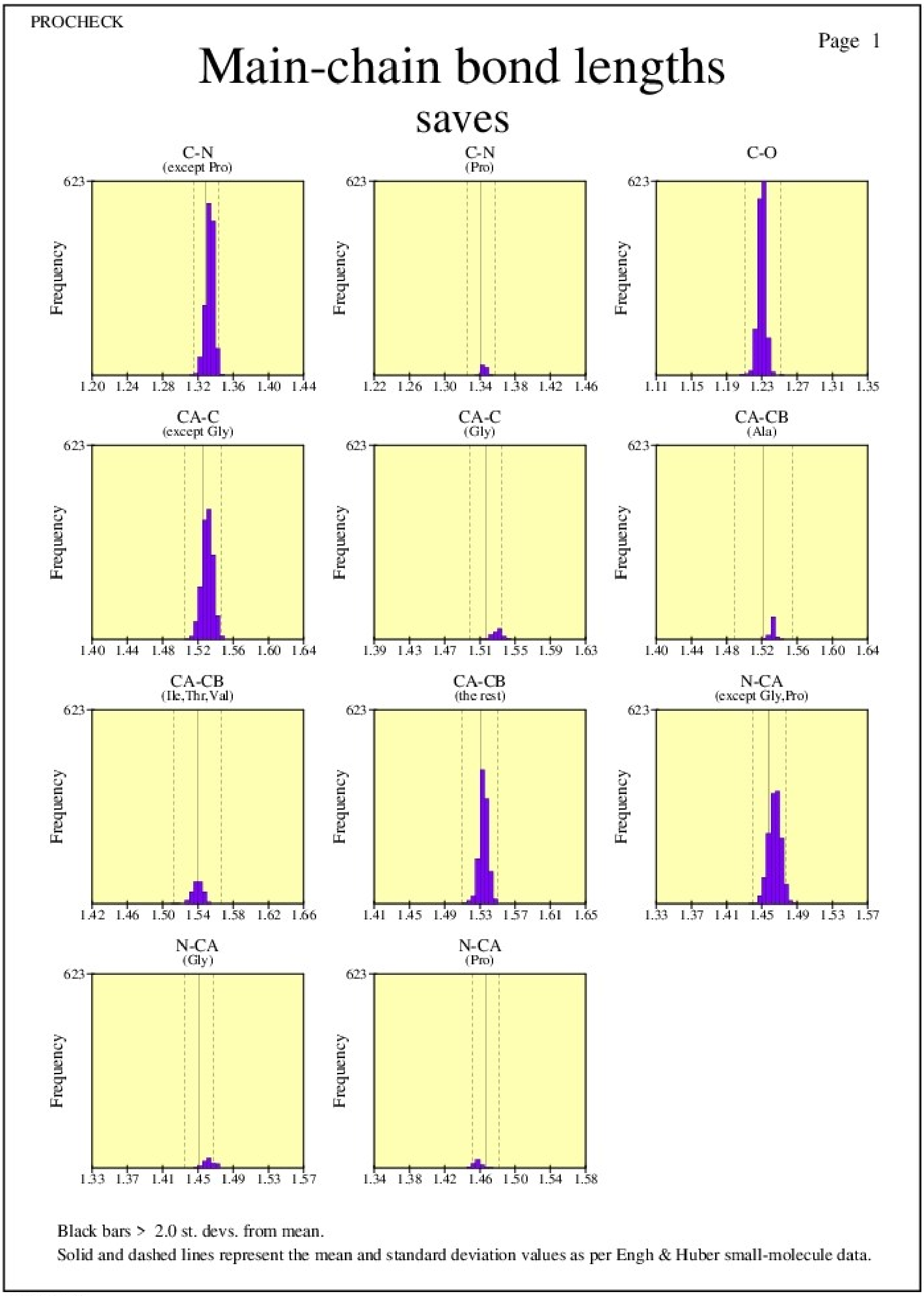

saves_07.ps

Figure S13: Main chain bond lengths of the Ro60-Ro52 complex obtained with PROCHECK

EXCLI Journal 2024;23:888-903 – ISSN 1611-2156
Received: January 24, 2024, accepted: April 26, 2024, published: June 10, 2024

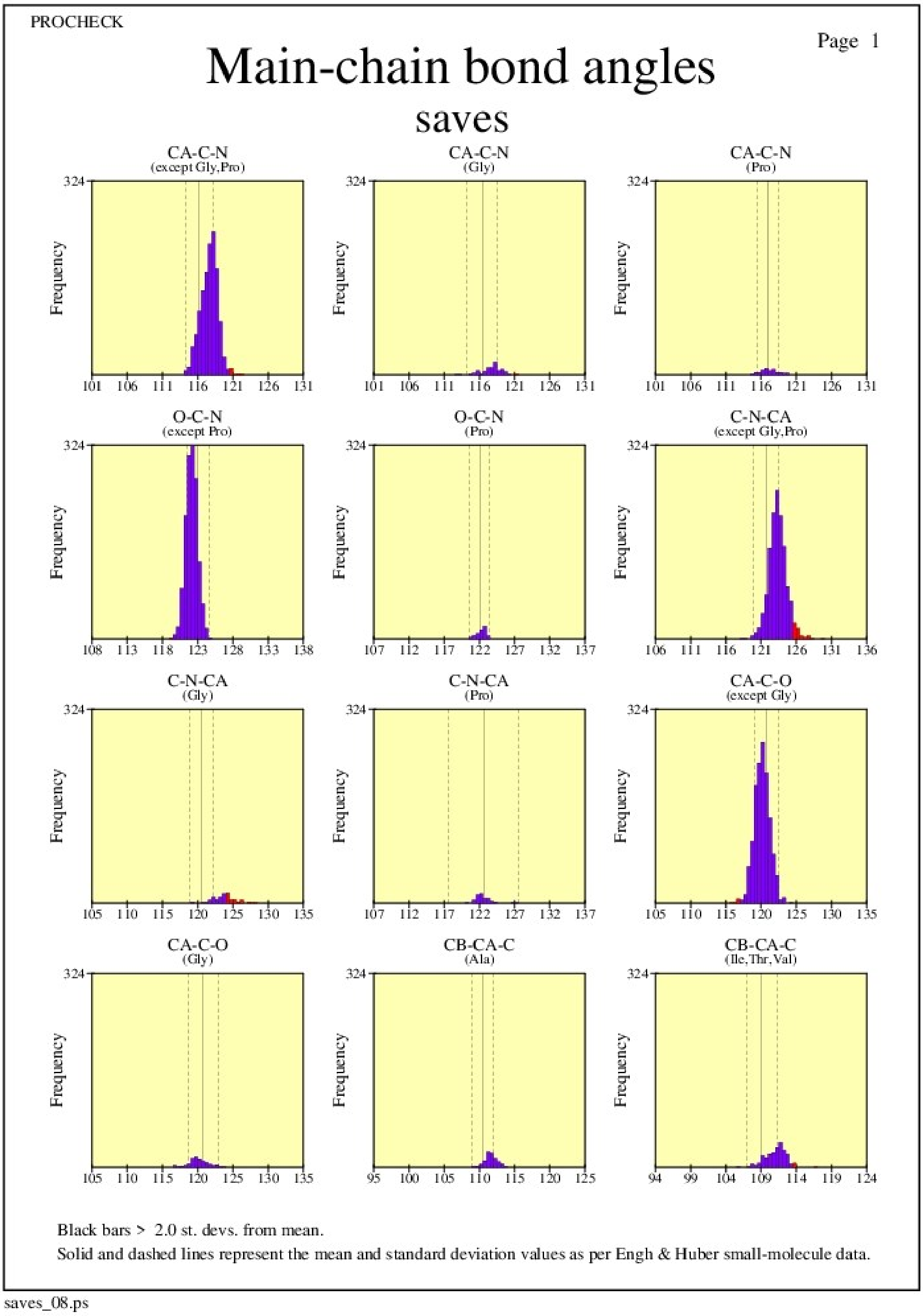

Figure S14: Bond angles of the main chain of the Ro60-Ro52 complex obtained with PROCHECK

EXCLI Journal 2024;23:888-903 – ISSN 1611-2156
Received: January 24, 2024, accepted: April 26, 2024, published: June 10, 2024

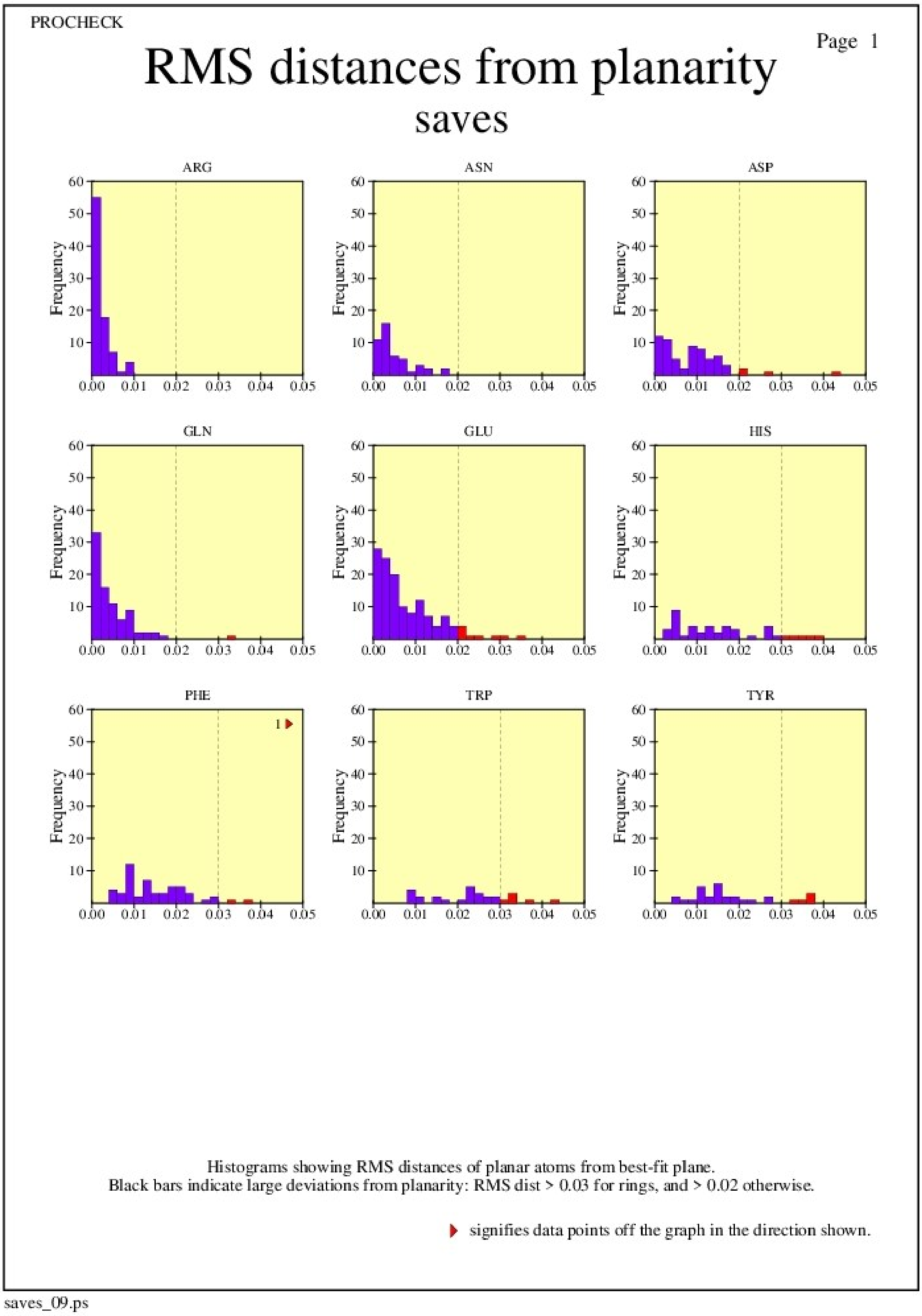

Figure S15: Planarity results of the Ro60-Ro52 complex obtained with PROCHECK

EXCLI Journal 2024;23:888-903 – ISSN 1611-2156
Received: January 24, 2024, accepted: April 26, 2024, published: June 10, 2024

Swiss-Model

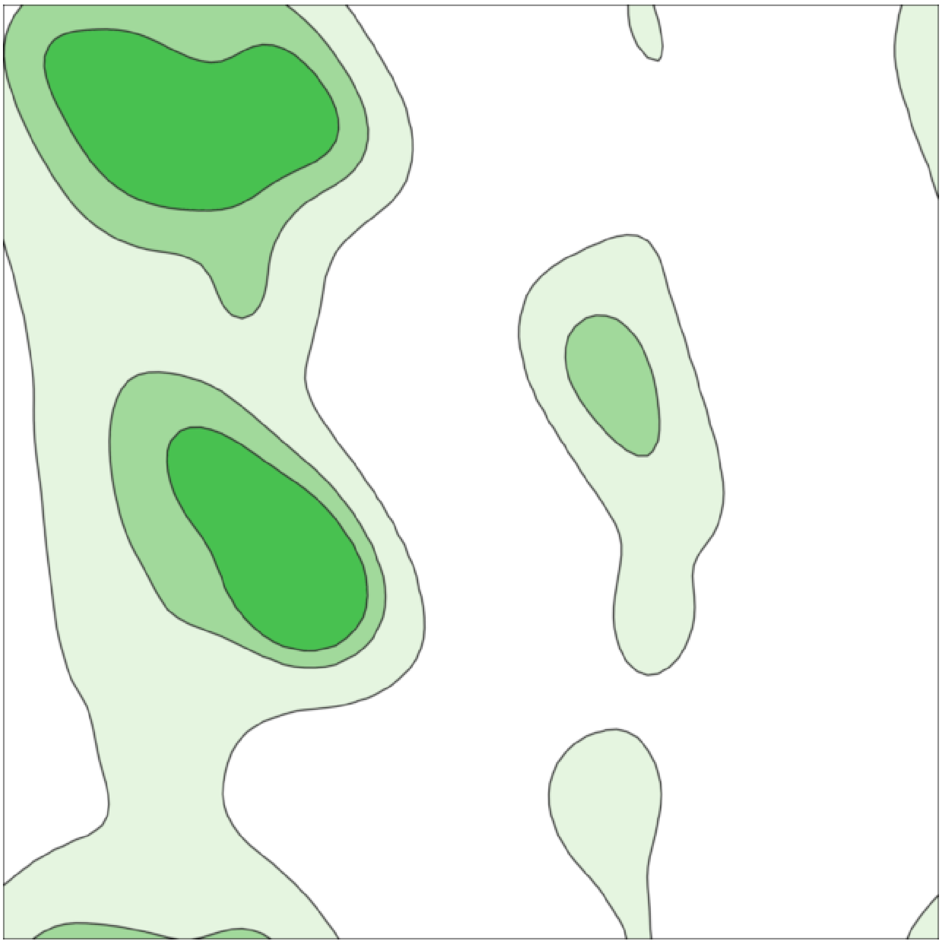

Figure S16: Ramachandran plot for the Ro60-Ro52 complex obtained with SWISS-MODEL Structure Assessment

EXCLI Journal 2024;23:888-903 – ISSN 1611-2156
Received: January 24, 2024, accepted: April 26, 2024, published: June 10, 2024

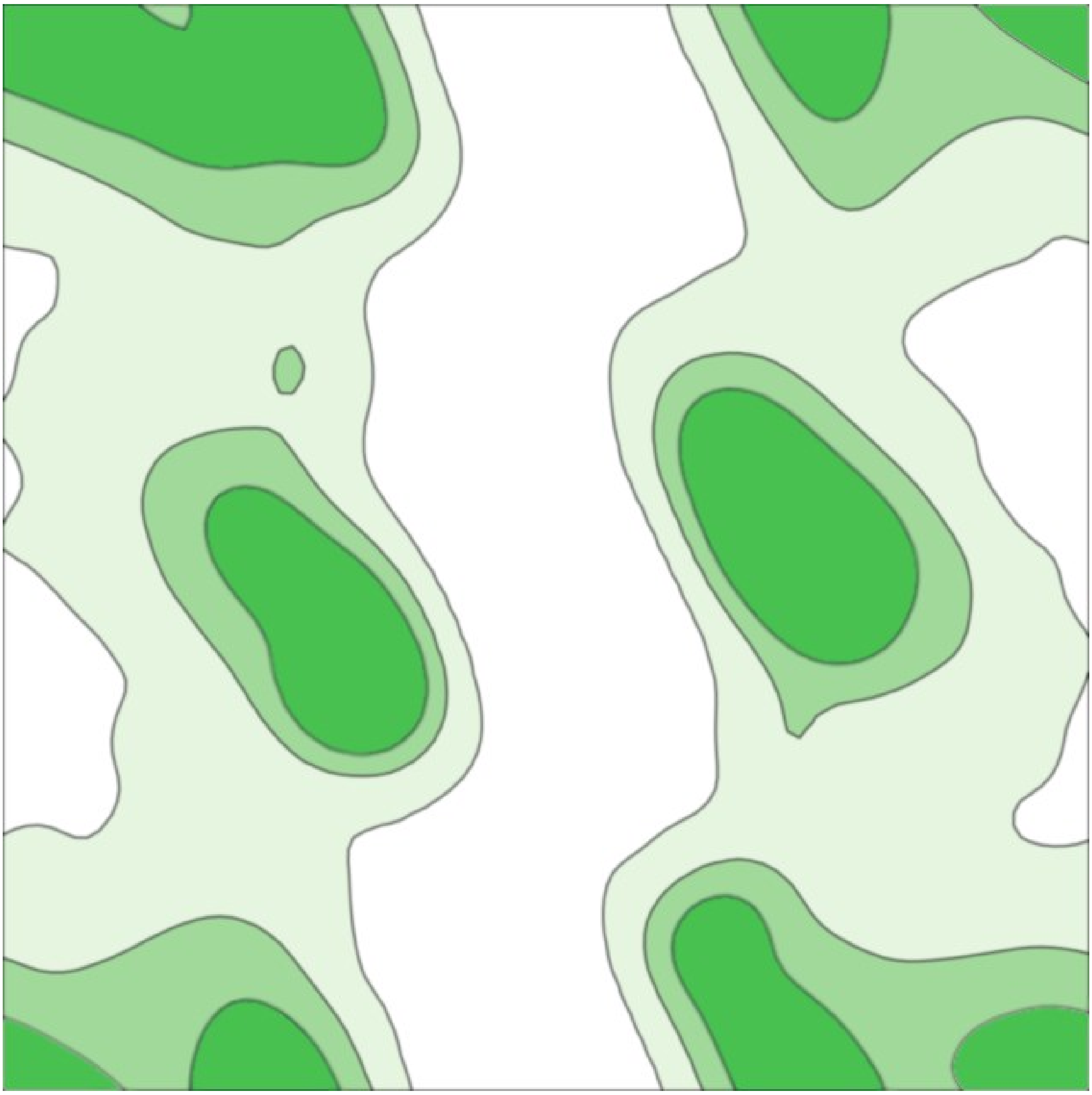

Figure S17: Ramachandran plot for the Gly residues of the Ro60-Ro52 complex obtained with SWISS-MODEL Structure Assessment

EXCLI Journal 2024;23:888-903 – ISSN 1611-2156
Received: January 24, 2024, accepted: April 26, 2024, published: June 10, 2024

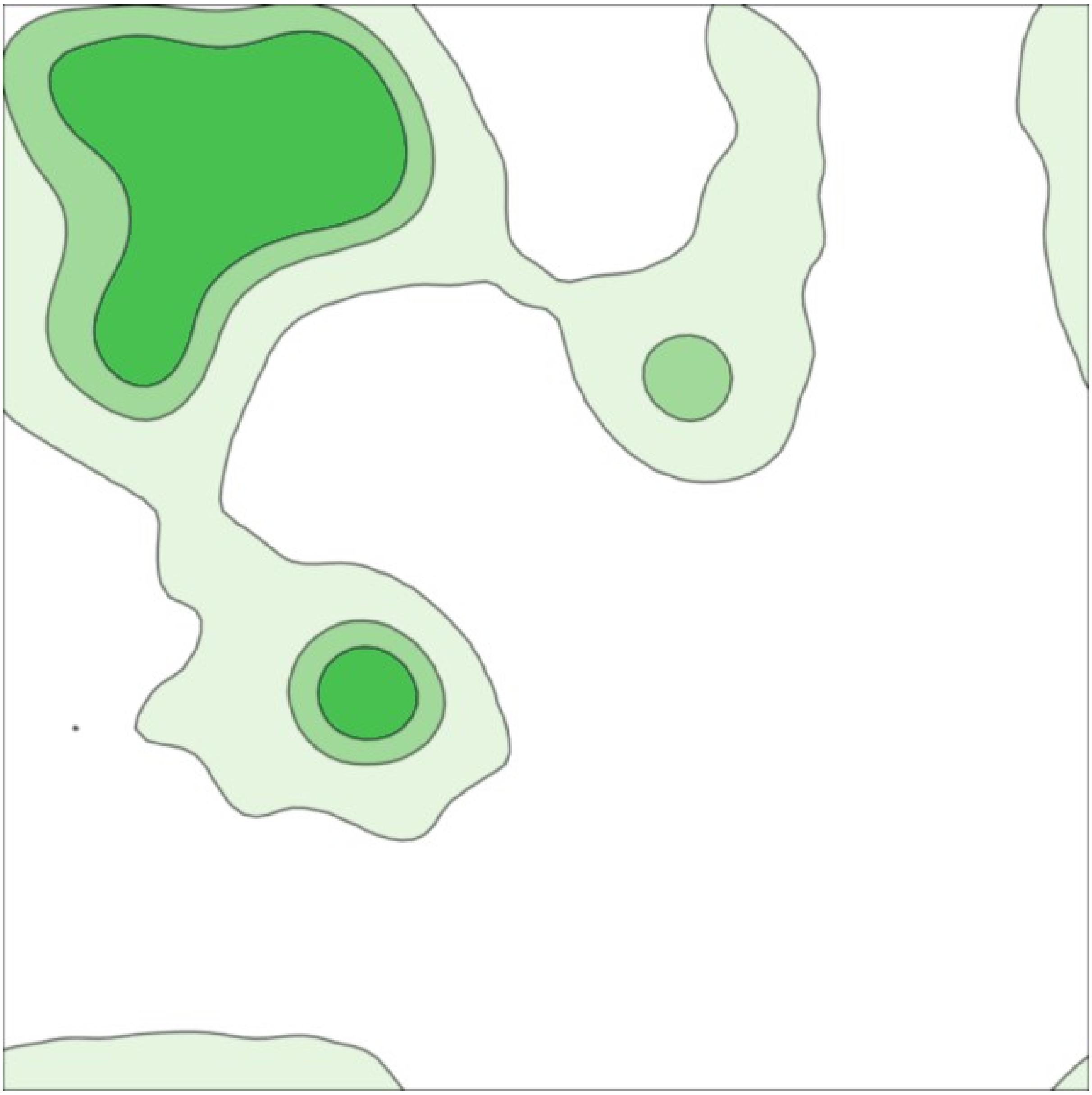

Figure S18: Ramachandran plot for the residues apearing before Pro in the Ro60-Ro52 complex obtained with SWISS-MODEL Structure Assessment

EXCLI Journal 2024;23:888-903 – ISSN 1611-2156
Received: January 24, 2024, accepted: April 26, 2024, published: June 10, 2024

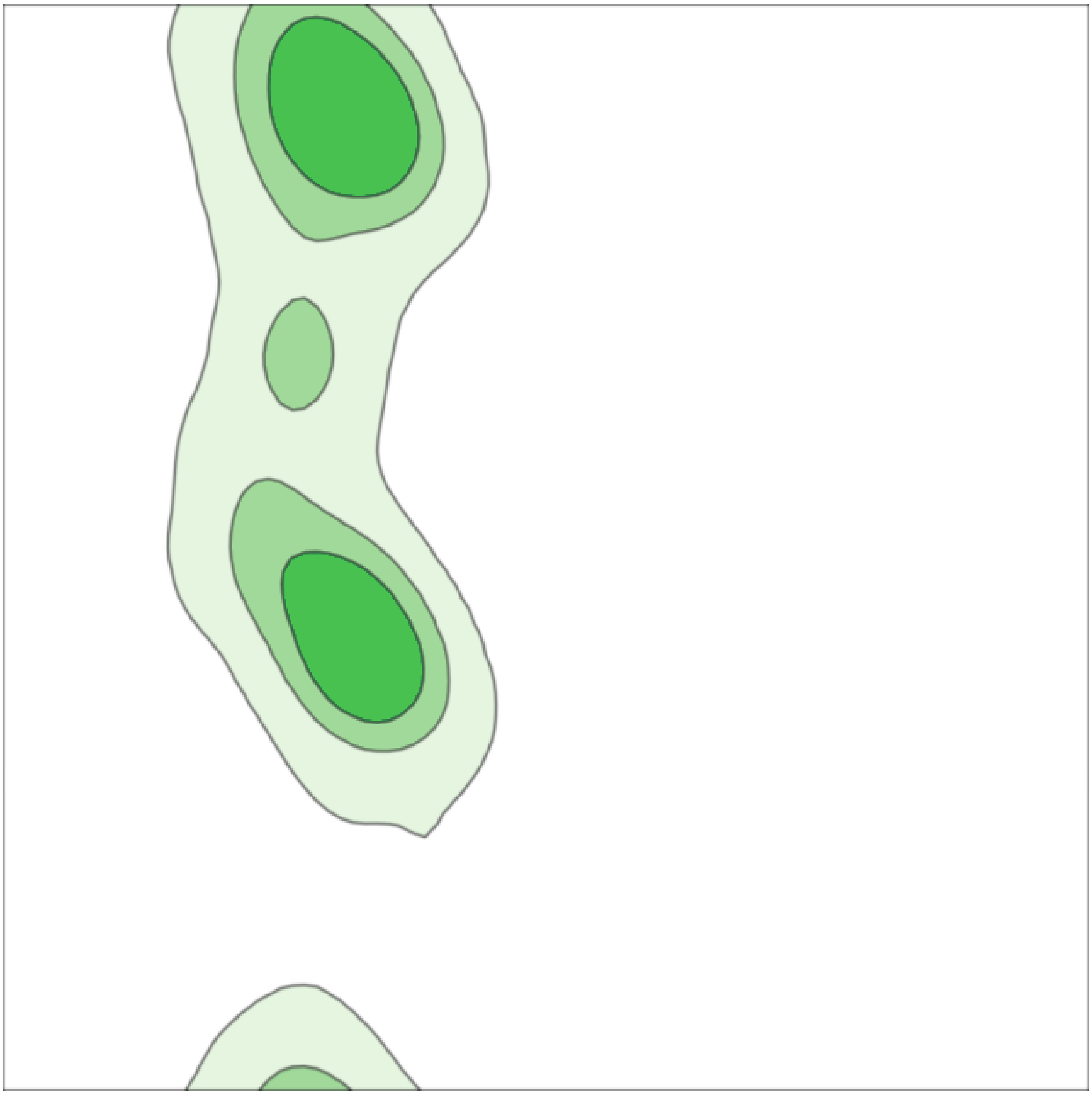

Figure S19: Ramachandran plot for the Pro residues of the Ro60-Ro52 complex obtained with SWISS-MODEL Structure Assessment

EXCLI Journal 2024;23:888-903 – ISSN 1611-2156
Received: January 24, 2024, accepted: April 26, 2024, published: June 10, 2024

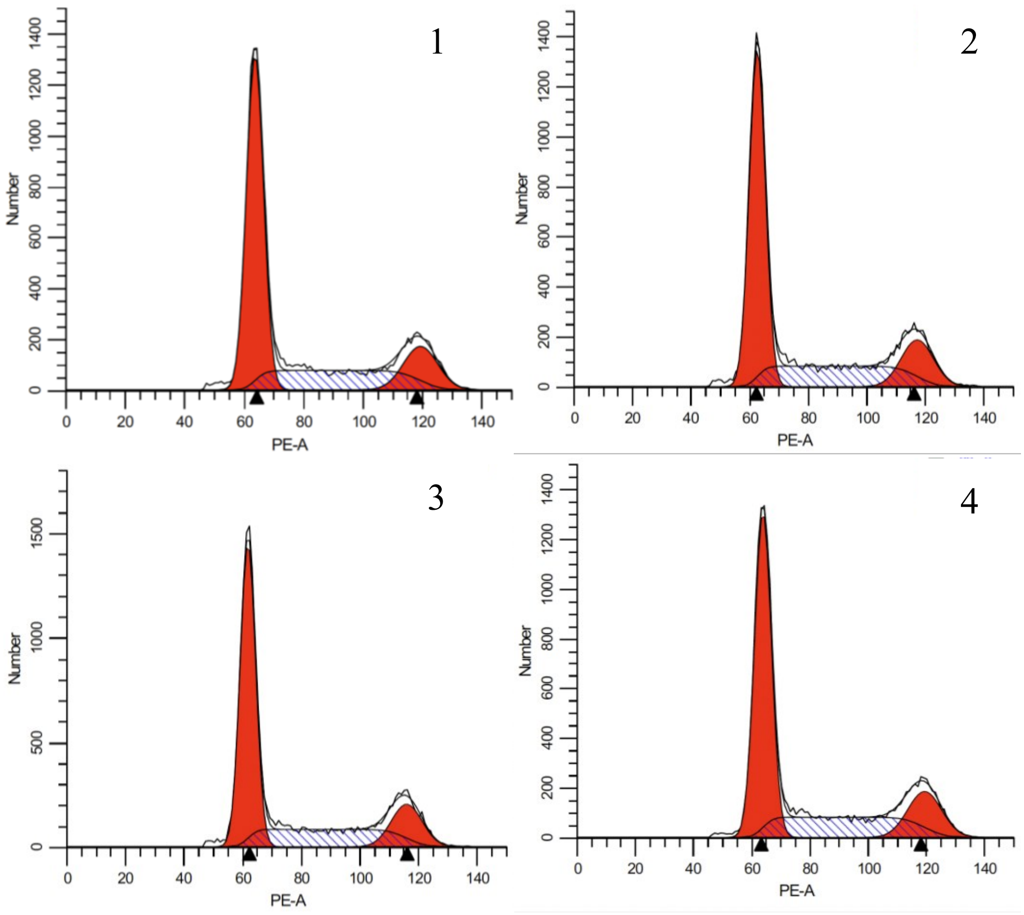

Cell Cycle	1	2	3	4	Mean	SD
G0/G1	58,84	56,95	57,20	56,94	57,48	0,91
S	25,96	27,30	26,81	26,99	26,77	0,57
G2/M	15,20	15,76	15,99	16,07	15,76	0,39

Figure S20: Analysis of the cell cycle in HeLa cells by Flow Cytometry. Each histogram represents one biological independent experiment (n=4). The table displays the results from the 4 experiments (numbered 1-4), the mean as well as the standard deviation (SD). The values refer to the percentage of cells in each of the phases of the cell cycle.
